# Effects of body plan evolution on the hydrodynamic drag and energy requirements of swimming in ichthyosaurs

**DOI:** 10.1098/rspb.2018.2786

**Published:** 2019-03-06

**Authors:** Susana Gutarra, Benjamin C. Moon, Imran A. Rahman, Colin Palmer, Stephan Lautenschlager, Alison J. Brimacombe, Michael J. Benton

**Affiliations:** 1 School of Earth Sciences, University of Bristol, Life Sciences Building, 24 Tyndall Avenue, Bristol BS8 1TQ, UK; 2 Oxford University Museum of Natural History, Parks Road, Oxford OX1 3PW, UK; 3 School of Geography, Earth and Environmental Sciences, University of Birmingham, Birmingham B15 2TT, UK

**Keywords:** ichthyosaurs, marine reptiles, marine tetrapods, swimming mechanics, fluid dynamics, CFD

## Abstract

Ichthyosaurs are an extinct group of fully marine tetrapods that were well adapted to aquatic locomotion. During their approximately 160 Myr existence, they evolved from elongate and serpentine forms into stockier, fish-like animals, convergent with sharks and dolphins. Here, we use computational fluid dynamics (CFD) to quantify the impact of this transition on the energy demands of ichthyosaur swimming for the first time. We run computational simulations of water flow using three-dimensional digital models of nine ichthyosaurs and an extant functional analogue, a bottlenose dolphin, providing the first quantitative evaluation of ichthyosaur hydrodynamics across phylogeny. Our results show that morphology did not have a major effect on the drag coefficient or the energy cost of steady swimming through geological time. We show that even the early ichthyosaurs produced low levels of drag for a given volume, comparable to those of a modern dolphin, and that deep ‘torpedo-shaped’ bodies did not reduce the cost of locomotion. Our analysis also provides important insight into the choice of scaling parameters for CFD applied to swimming mechanics, and underlines the great influence of body size evolution on ichthyosaur locomotion. A combination of large bodies and efficient swimming modes lowered the cost of steady swimming as ichthyosaurs became increasingly adapted to a pelagic existence.

## 1. Introduction

Ichthyosaurs were an iconic group of marine reptiles that lived from the Early Triassic to the early Late Cretaceous (*ca* 248–93.9 Ma) [1–5]. Note that we adopt the common term ‘ichthyosaur’ in a broad sense to refer to all ichthyosauriform taxa [1]. The earliest ichthyosaurs were characterized by lizard-shaped, flexible bodies and elongate tails with either no distinctive caudal fin or low-aspect-ratio heterocercal ones [6,7]. By the Jurassic, they had evolved deep-bodied, fish-like morphologies, with increasingly differentiated caudal vertebrae and high-aspect-ratio lunate flukes [8], adaptations associated with a switch from anguilliform (i.e. eel-like) to carangiform (i.e. mackerel-like) swimming [7,8]. This transition to a more streamlined body shape, as seen in modern fast cruisers such as tuna, dolphins and lamnid sharks, may also have reduced their drag, thereby potentially enhancing locomotory performance and optimizing the energy balance of swimming [9,10]. However, despite some work exploring the relationship between morphology and functional performance in fossil marine reptiles [6,7,11], the impact of body shape on the hydrodynamic properties and energy cost of swimming in ichthyosaurs is not well understood.

A simplified approach to studying the energetic balance of steady swimming uses a model in which the animal is represented by a rigid body that moves through the water at a constant speed overcoming drag. Swimming is an unsteady phenomenon, and drag is affected by several factors such as body flexibility and kinematics [12,13]. Nevertheless, this model allows us to focus on the contribution of morphology to drag while minimizing assumptions about swimming kinematics. Previous research on the drag of ichthyosaurs used methods based on empirically derived formulae, approximating ichthyosaur bodies to idealized ellipsoid forms [6,11]. Many ichthyosaurs departed greatly from these simple shapes, especially the earliest species [1,14]. Here we use three-dimensional modelling tools to produce more detailed representations of the animals' geometries in order to investigate the effects of morphology on the drag coefficient and the cost of locomotion (i.e. energy spent transporting a unit mass per unit distance).

**Figure 1.**
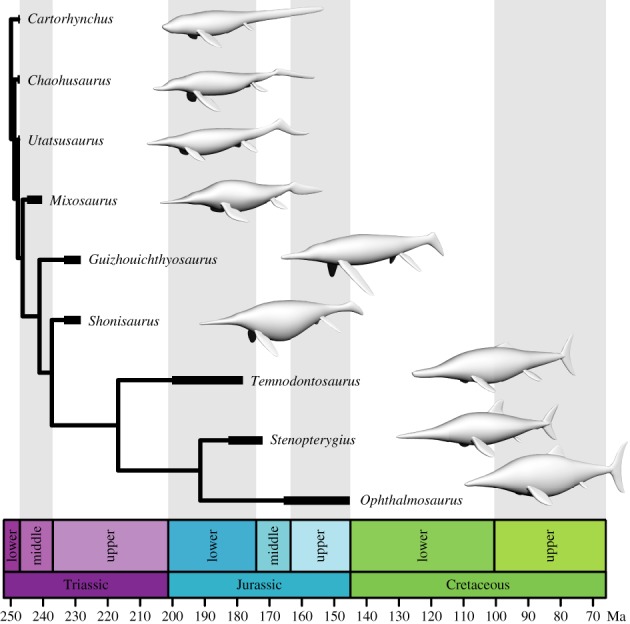
Digital models of the ichthyosaurs analysed in this study shown in their phylogenetic context. Simplified phylogeny modified after reference [19]. All models scaled to the same length. (Online version in colour.)

Knowledge of ichthyosaur body forms has been improved by the discovery of several complete specimens in the past decade [15–17], including important basal taxa [1,14]. Moreover, recent systematic work has provided a comprehensive phylogenetic framework for ichthyosaurs [2,18,19]. Taking advantage of this, we created three-dimensional models of nine ichthyosaurs known from well-preserved fossil specimens (figure 1 and electronic supplementary material, figure S1A). The taxa selected occupy a wide range of phylogenetic positions and are representative of the main body shapes and sizes of ichthyosaurs, an advance relative to former studies, which focused only on derived forms [6,11]. Using computational fluid dynamics (CFD) [20], a numerical technique for simulating fluid flows, we tested the hypothesis that the derived fish-shaped ichthyosaurs had acquired morphologies that reduced the energy cost of steady swimming.

## 2. Material and methods

### (a) Three-dimensional modelling

Nine ichthyosaurs were selected on the basis of excellent preservation and completeness, permitting the construction of three-dimensional models (see the electronic supplementary material, methods and figure S1A): the Early Triassic *Cartorhynchus lenticarpus, Chaohusaurus geishanensis* and *Utatsusaurus hataii* are referred to as ‘basal grade’; the Middle and Late Triassic *Mixosaurus cornalianus*, *Shonisaurus popularis* and *Guizhouichthyosaurus tangae* are grouped into the ‘intermediate grade’; and post-Triassic *Temnodontosaurus platyodon*, *Stenopterygius quadriscissus* and *Ophthalmosaurus icenicus* are identified as ‘fish-shaped ichthyosaurs', adopting a nomenclature proposed elsewhere [21] for the three main ichthyosaur morphotypes. A model of the extant bottlenose dolphin *Tursiops truncatus* was also included in the analysis (see the electronic supplementary material, methods). In all cases, models were constructed both with and without limbs. To assess the precision of the modelling technique, measurements of surface area and mass from the *Tursiops* model were compared to those reported for live specimens (electronic supplementary material, figure S1B), and were found to be a very good approximation. Consequently, we inferred that our three-dimensional models could be used to estimate these parameters accurately [22,23]. The specimens under study exhibit a wide range of sizes, from the small *Cartorhynchus* (under 50 cm in length) to the colossal *Shonisaurus* (more than 12 m in length). Hence, we created a battery of models scaled to a total length of 1 m (electronic supplementary material, table S1) that would serve to compare all body shapes controlling for the differences in size. A ZIP file containing the three-dimensional models used in this research can be downloaded from the Dryad Digital Repository: https://doi:10.5061/dryad.n222q81 [24].

### (b) Computational fluid dynamics

**Figure 2.**
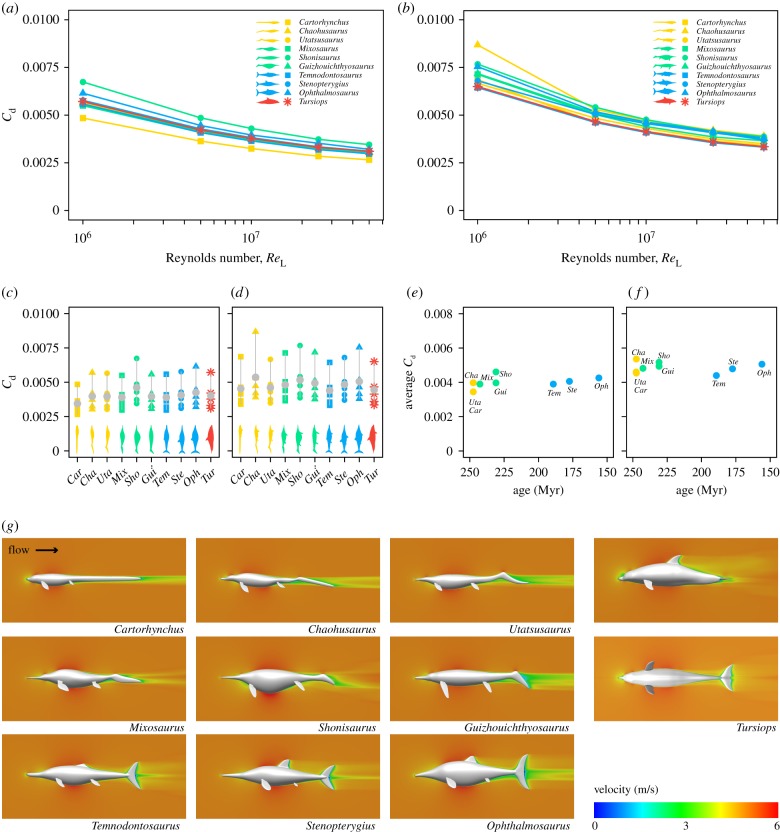
Drag coefficients of nine ichthyosaurs and a modern analogue, the bottlenose dolphin. (*a*,*b*) CFD-computed total drag coefficients of nine ichthyosaurs and a bottlenose dolphin without (*a*) and with (*b*) limbs at Reynolds numbers from 10^6^ to 5 × 10^7^. (*c*,*d*) Comparison of the drag coefficients and their mean values (in grey) between taxa, without (*c*) and with (*d*) limbs; two-sample *t*-tests between groups not significant (NS). (*e*,*f*) Mean values of the drag coefficient of ichthyosaurs plotted against the mean occurrence age for each taxon, without (*e*) and with (*f*) fins; no correlation detected, Kendall's *τ* = −0.29, *p* = 0.28, NS (no limbs); Kendall's *τ* = −0.22, *p* = 0.39, NS (with limbs). Ichthyosaurs from the ‘basal grade’ are highlighted in yellow, the ‘intermediate grade’ in green and the ‘fish-shaped ichthyosaurs' in blue. The bottlenose dolphin *Tursiops* is highlighted in red. (*g*) Two-dimensional plots of flow velocity magnitude (*Re* = 5 × 10^6^; inlet velocity of 5 m s^−1^).

CFD analyses were carried out using ANSYS-Fluent (v. 18.1 Academic). For each model of length *L*, a computational domain was created consisting of a cylinder with a length of 10 × *L* downstream, 3 × *L* upstream and a radius of 5 × the maximum width of the model (electronic supplementary material, figure S1C). As these models are bilaterally symmetrical, only half of the model geometry and half of the enclosing cylinder were used in order to economize computational resources. A normal inflow velocity inlet was defined at the upstream end of the cylinder and a zero-pressure outlet at the downstream end. Symmetry boundary conditions were assigned to the sides of the cylinder to model a zero-shear wall, whereas the walls of the model itself were assigned a no-slip boundary condition, constraining the fluid velocity at zero relative to the model. The domain was meshed using a combination of tetrahedral and prismatic mesh elements (see the electronic supplementary material, methods). Because the Reynolds numbers (*Re*) of the simulations fall within the turbulent flow regime (*Re* > 10^6^), the shear stress transport turbulence model was used to solve the Reynolds-averaged Navier–Stokes equations (see the electronic supplementary material, methods). A double precision, stationary pressure-based solver and a second-order discretization method were used to compute the steady-state flow patterns. Convergence (i.e. the moment when the iterative simulation process reaches a stable solution) was judged on the basis of a stable numerical solution for the integrated value of drag, root-mean-square residual levels of 10^−4^, and a mass flow rate imbalance smaller than 1%. The results were visualized as false-colour contour plots of flow velocity magnitude (figure 2*g* and electronic supplementary material, figure S2B) and pressure coefficients (electronic supplementary material, figure S3E). In addition, the total drag forces (*D*) were extracted and the drag coefficients were calculated $(C_{d}=2D\;/\;\rho \;u^{2}S$; where *ρ* is the density of water, 998.2 kg m^−3^ at 20°C; *u* is the inlet velocity in m s^−1^ and *S* is the wetted surface area of the model in m^2^). The internal components of drag (i.e. skin friction, *D*_f_, and pressure drag, *D*_p_) were also extracted, and their respective coefficients calculated in the same manner (electronic supplementary material, data S1). The CFD methodology used herein was validated against existing experimental data from water tank experiments (see the electronic supplementary material, methods and figure S2).

For the CFD simulations of the ichthyosaurs and dolphin, various inlet velocities were applied corresponding to Reynolds numbers from 10^6^ to 5 × 10^7^, to encompass the range of *Re* observed in extant swimming tetrapods of similar dimensions [9]. To eliminate the effect of size, we computed the drag for length-scaled geometries at the same speed (i.e. dynamic similarity, given by equivalent Reynolds numbers [10]). Additional calculations were carried out for geometries scaled to the same volume (i.e. same mass, assuming a uniform density) and life-size dimensions. For the life-size analyses, we used body length values documented for each genus in the literature (electronic supplementary material, data S3). All simulations were performed with the models scaled to a total length of 1 m, with *Re* adjusted for each analysis, as the drag of a given geometry at a specific *Re* corresponds to an infinite number of combinations of length and velocity [25]. The models were scaled to the specified dimensions in Rhinoceros (v. 5.5.3) to obtain the geometric variables.

### (c) Drag per unit volume and net cost of locomotion

Drag per unit volume represents the proportion of drag power to maximum muscular power available for locomotion. Moreover, the drag-to-volume ratio is a proxy for the cost of locomotion dedicated to overcoming drag during steady swimming, as outlined below. The cost of transport (COT) is the mass-specific energy spent over a unit distance [26] and the net or mechanical cost of transport (COT_net_) is the fraction of COT exclusively dedicated to locomotion, which excludes the basal metabolism and the losses owing to muscle efficiency [27]. COT_net_ is calculated as the mechanical power output (*P*_out_) divided by the mass (*m*) and the speed (*u*). The ratio of useful power (thrust power, *P*_thrust_, equal to drag power, *P*_drag_, at constant speed) to *P*_out_ is the propulsive efficiency (*η*). We can therefore express COT_net_ in terms of the drag power:

2.1 $$\begin{array}{c} \mathrm{CO}T_{\mathrm{net}}=\frac{P_{\mathrm{out}}}{m\;u}=\frac{\;P_{\mathrm{drag}}}{\eta \;m\;u} \end{array}$$

The contribution of the drag to the net cost of locomotion, here termed COT_drag_, is proportional to the drag per unit volume:

2.2 $$\begin{array}{c} \mathrm{CO}T_{\mathrm{drag}}=\frac{P_{\mathrm{drag}}}{m\;u}\;=\frac{D}{\rho \;V} \end{array}$$

where *V* is the animal's volume and *ρ* is its density.

**Figure 3.**
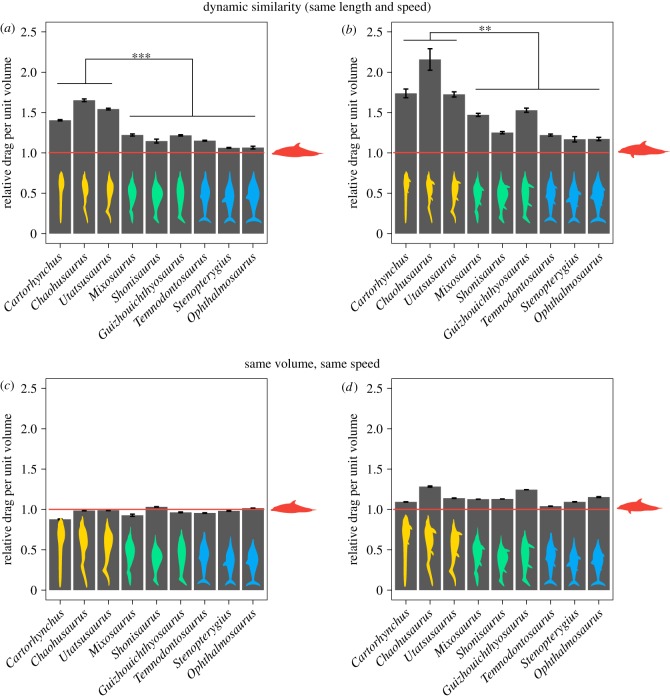
Relative drag per unit volume of ichthyosaurs. (*a*,*b*) Normalized drag per unit of volume without (*a*) and with (*b*) limbs, for nine ichthyosaurs scaled to the same total length and tested at the same speed, relative to the values for a bottlenose dolphin (mean ± s.d.). Two-sample *t*-test ***p = 0.00044 (no fins), **p = 0.0037 (full body) between the ‘basal grade' and the rest of ichthyosaurs. (*c*,*d*) Normalized drag per unit of volume without (*c*) and with (*d*) limbs for nine ichthyosaurs scaled to the same volume and tested at the same speed, relative to the values for a bottlenose dolphin (mean ± s.d.). No significant differences detected between ichthyosaur grades.

We divided the computed drag by the volume of each model at various hypothetical combinations of body length (1, 2 and 10 m) and velocity (from 1 to 5 m s^−1^) for the models scaled to total length (electronic supplementary material, table S2), encompassing sizes observed in ichthyosaurs and velocities that are likely to occur in living aquatic and semiaquatic animals of those dimensions [9]. The same calculation was performed for volume-scaled models, at a velocity of 1 m s^−1^ (electronic supplementary material, table S3). All the results were then normalized relative to the bottlenose dolphin (figure 3).

We then evaluated the importance of body shape in relation to other factors known to affect the cost of locomotion, namely the swimming mode and the body size. For this, we calculated the COT_net_ for each taxon using the full length-scaled, volume-scaled and life-size models, assuming no differences according to swimming mode (i.e. adopting a propulsive efficiency of *η* = 1 for all taxa), as well as incorporating estimates of propulsive efficiency for undulatory swimming in extant aquatic animals (electronic supplementary material, data S2). Results were also normalized relative to the values obtained for the dolphin. We adopted *η* = 0.73–0.91 (average of 0.81) for the dolphin, based on published estimates [28,29]. The swimming modes of ichthyosaurs are thought to range from anguilliform in the earliest forms, to carangiform/thunniform in the most derived ichthyosaurs [7]. Jurassic and younger ichthyosaurs typically show a demarcated tailbend, indicative of a crescent tail fin [21,30]. Hence, we assumed that the post-Triassic ichthyosaurs were carangiform/thunniform swimmers, and therefore assigned them the dolphin's swimming efficiency. All the non-parvipelvian ichthyosaurs were assumed to be closer to the anguilliform end of the spectrum and were assigned values of swimming efficiency measured in extant eels, *η* = 0.43–0.54 (average of 0.48) [31]. The intermediate forms included the anguilliform swimmers based on their high presacral vertebral counts, which point towards flexible backbones, and their caudal morphology, showing less conspicuous tailbends or absence thereof [15–17]. These simple assumptions, also adopted by previous studies [6], allow us to incorporate the potential effects of kinematics on the energy requirements of steady swimming in our models. This paper is not concerned with the absolute values of *η* or the differences between individual taxa, but with the relative impact of the shift of swimming mode on COT_net_ compared to the relative effect of body shape. Efficiency estimates from dynamic flow simulations show differences between anguilliform and carangiform of a similar order of magnitude [32,33].

## 3. Results

### (a) Effect of body plan on the drag coefficient of ichthyosaurs

Validation experiments demonstrate that the CFD simulations can replicate the experimental drag coefficients of various torpedo-like forms within less than 5% error (electronic supplementary material, figure S2A–C), accurately capturing small variations in drag owing to the different fineness ratios (FR: the ratio between total length and maximum diameter). This confirms that our simulation methodology can be used to compute drag forces accurately for three-dimensional objects, establishing the validity of the approach.

The drag coefficients of the ichthyosaurs both with and without limbs (*Re* = 10^6^–5 × 10^7^) did not change substantially between the ichthyosaur morphological grades or through geological time (figure 2*a–f*). We present velocity plots (figure 2*g*) and pressure distributions (electronic supplementary material, figure S3E), which show features such as the stagnation point at the tip of the rostrum, the flow acceleration around the body's maximum diameter and a low velocity wake, with broadly similar patterns in all taxa. We assume smooth, fully turbulent flow, consistent with the current evidence that suggests a mainly turbulent boundary layer in animals swimming in transitional regimes, like dolphins [34,35]. This also acknowledges that skin roughness, a factor that influences the extent of laminar flow, is usually not preserved in fossils. In all cases, the skin friction was recovered as the main component of drag (electronic supplementary material, figure S3A–D), as expected of slender streamlined bodies [25], with values very close to the empirical formula for turbulent skin friction ITTC 57 [36].

The changes in drag coefficient owing to the shape of the trunk alone are small, on average less than 5% when the ichthyosaurs are compared to each other and to the bottlenose dolphin (figure 2*a,c*). *Cartorhynchus* and *Shonisaurus* are an exception to this, with body forms that produce 15% higher and 15% lower drag coefficients, respectively, compared to *Tursiops*; these extreme values are mainly caused by differences in the pressure drag (electronic supplementary material, figure S3C,D). Simulations of the full morphology (figure 2*b*,*d*) produce higher drag coefficients than the trunk with no limbs. This is owing, in part, to the interference effects between the limbs and the body (i.e. interference drag), which are captured by CFD and would otherwise be impossible to predict with empirically derived formulations [37]. The drag coefficients of the full morphology also show a greater range, revealing that differences between taxa are larger when considering the full body and appendages. The average contribution of the limbs to the total drag coefficient is about 24% for the ichthyosaurs and only about 10% in *Tursiops*, differences that are associated with the relatively larger limbs of ichthyosaurs compared to the dolphin (electronic supplementary material, table S1), and the absence of hindlimbs in the latter. Overall, there are no significant changes in drag coefficient owing to body shape associated with morphotype (two sample *t*-test, *p* > 0.05) or geological time (Kendall's tau, *p* > 0.05) (figure 2*c*–*f*).

### (b) Drag per unit volume of ichthyosaurs and net cost of locomotion

Scaling to length or volume produces different patterns of drag per unit volume across the taxa under study. For a constant total length, the basal-grade ichthyosaurs generate on average 1.4 times more drag per unit volume for all combinations of velocity and size tested, compared to the intermediate and fish-shaped grades (figure 3*a*,*b* and electronic supplementary material, table S2). This difference is significant when testing the trunk only (*t*-test: *p* < 0.001 for the data with no limbs) as well as the full morphology (*t*-test: *p* < 0.01 for the data with limbs). Under these conditions, the bottlenose dolphin has the lowest drag per unit volume in all cases, followed closely by the parvipelvian *Ophthalmosaurus*, while the highest values are found in *Chaohusaurus.* On the other hand, when scaling to volume, there are no significant differences between grades, with the ichthyosaurs producing values close and sometimes lower than the dolphin (figure 3*c*,*d* and electronic supplementary material, table S3).

As expected for slender bodies, skin friction is the main drag component in our ichthyosaur models, and thus, total drag scales roughly with surface area. Therefore, under length-scaling, high drag per unit volume is observed in animals with large FR and high surface-to-volume ratios (electronic supplementary material, figure S5A–D). By contrast, volume-scaled models have rather uniform surface area-to-volume ratios (electronic supplementary material, figure S5F,H). Additionally, under volume scaling, we do not observe a clear correlation between FR and drag (electronic supplementary material, figure S5E,G). As with the drag coefficient, the limbs increase the drag per unit volume relative to a limbless body to varying degrees. Of all models tested, the lowest proportion of drag because of the limbs is for *Tursiops*, which has only two relatively small flippers, while the largest drag is for *Chaohusaurus* and *Guizhouichthyosaurus*, with four relatively large flippers. Although this contribution can be substantial in some forms, it does not change the overall trend between morphotypes.

**Figure 4.**
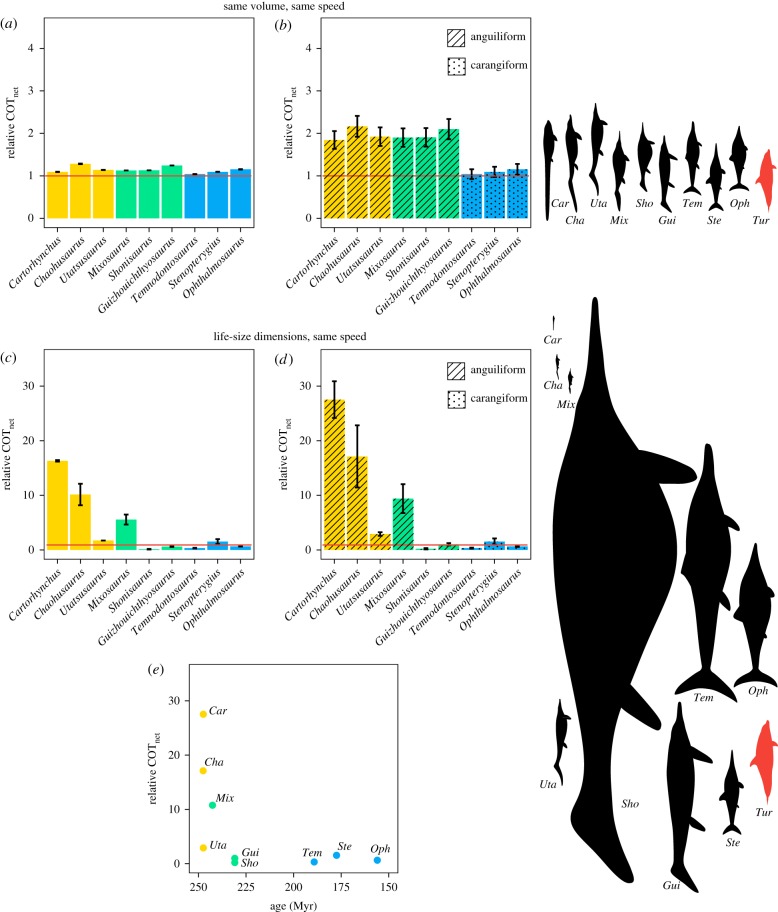
Comparison of the effects of body shape, swimming style and body size on the net energy cost of steady swimming in ichthyosaurs. (*a*,*b*) Relative net cost of steady swimming (COT_net_) for ichthyosaurs of the same mass moving at the same speed. (*a*) Differences owing to morphology, not accounting for swimming style (propulsive efficiency, *η* = 1). (*b*) Differences owing to body shape and swimming style, incorporating propulsive efficiency estimates from living aquatic vertebrates; *η* = 0.48 for anguilliform swimmers [31] and *η* = 0.81 for carangiform swimmers [28,29]. (*c*,*d*) Relative differences in the net cost of swimming owing to body shape and size (length for each taxon is the mean of multiple specimens), moving at the same speed of 1 m s^−1^, when swimming efficiency is not accounted for (*η* = 1) (*c*), or (*d*) after incorporating the propulsive efficiency as in (*b*). (*e*) Mean COT_net_ of ichthyosaurs at life-size scale calculated as in (*d*), plotted against the mean occurrence age for each taxon. Colour coding for (*a*–*e*) corresponds to the one used in figures 2 and 3.

Cost of locomotion is usually represented relative to body mass [9,10]. Consistent with this, we adopt the volume (a mass proxy) as the most biologically relevant parameter for comparing energetic performance [9]. Our results suggest that the change in body plan did not have an impact on the net cost of steady swimming (figure 4*a*). Accounting for propulsive efficiency illustrates that, in the absence of morphological effects, the relative differences in COT_net_ between ichthyosaurs for a given volume (figure 4*b*) would come down to swimming mode. Based on efficiency estimations made in living aquatic animals [28,29,31], carangiform swimming can reduce up to 50% the COT_net_ relative to anguilliform swimming at steady, high speeds (i.e. high *Re*, inertial regime, consistent with the *Re* of our experiments). Finally, our results show that size has the largest impact on the COT_net_ of steady swimming (figure 4*c*,*d*). A doubling in length can reduce the drag to volume ratio by about 55% and this reduction can be as much as 85% if the length increases by a factor of 5 (electronic supplementary material, table S2 and data S2), which happens because surface area, and hence drag, increases with length squared, whereas volume increases with length cubed. The experiments with life-sized ichthyosaurs show that differences in the COT_net_ between basal and derived ichthyosaurs are exacerbated by their differences in size (figure 4*c*,*d*). At 1 m s^−1^, a 40 cm *Cartorhynchus* would incur 24 times higher COT_net_ than a 4 m *Ophthalmosaurus*, if swimming mode is not considered, and about 42 times higher if swimming mode is accounted for. We observe a clear reduction in the net cost of locomotion through ichthyosaur evolution, especially during the first 25 Myr, after which time the values remain relatively constant (figure 4*e*), a trend that is dominated by body size.

## 4. Discussion

Our computed drag coefficients for the bottlenose dolphin are consistent with those reported in the literature for gliding dolphins, rigid models and static CFD simulations of dolphins [35,38], although, as expected, they fall below estimates obtained from thrust-based methods (e.g. hydromechanical models) [28,29] because the simulations used herein do not account for the dynamic effects of drag [12,13,29]. The interaction between morphology and kinematics is not yet fully understood, and ideally, hydrodynamic modelling should integrate motion [13]. However, three-dimensional dynamic CFD is still computationally expensive and would require a large set of assumptions regarding the kinematics and the geometry of the propulsor elements of ichthyosaurs. Morphology alone has an undeniable effect on drag, as shown by a wealth of aerodynamics research [25,39,40]. Focusing on this, we employed static CFD as the most accurate tool for testing such a wide sample of animals. Additionally, it provides a good model for underwater gliding (i.e. inertial motion without body deformation), an energy-saving mechanism used by many aquatic animals during swimming or diving [41]. This study represents an important methodological advance relative to previous research, which estimated drag using empirical formulae based on axisymmetrical bodies for a small number of taxa [6,11].

We scaled our models to equal total length and equal volume to estimate the drag per unit volume in ichthyosaurs, both of which are valid scaling criteria to study the hydrodynamic effects of morphology. The former is often the choice for hydrodynamic studies [32,39], because controlling for dynamic similarity avoids the effect of Reynolds number on drag (*C*_d_ is smaller at larger *Re*) [10]. The latter is used in underwater vehicle research to look for designs with minimum drag for a given load [40,42,43]. In general, results of volume-scaled comparisons cannot be fully ascribed to morphology, because of the *Re* effect [32]; however, this only has a minor impact here, affecting the drag coefficient by less than 10%. Body mass determines key aspects of an animal's physiology and energetic balance [9,10], and thus volume (proxy for body mass) is the best normalizing parameter to compare energetic performance, which leads us to reject the hypothesis that a change in body plan reduced the cost of steady swimming in ichthyosaurs.

Classic experiments on volume-scaled slender rotational bodies are a recurrent reference when discussing the drag of aquatic animals [42,44]. Based on these, an FR close to 4.5 is often taken as an indication of optimal drag reduction. Our volume-scaled results agree with these experiments in that drag variation is small for a wide range of FR (FR in our models spans from 4.4 to 9); however, the differences we obtain are larger than the expected 10%, especially when the limbs are included, and there is no relationship between FR and drag. Thus, contrary to the general perception, an FR of 4.5 does not necessarily predict the lowest drag in aquatic animals, a point clearly illustrated by the experiments on limbless bodies (electronic supplementary material, figure S5E; *Cartorhynchus* has the lowest drag, with an FR = 8.4). FR can only predict drag for a given volume when all other geometric parameters are constant, which is not the case for complex organic shapes. We also show that the size and morphology of the limbs have an effect on the total drag (electronic supplementary material, figure S5). The modification of flow by appendages and control surfaces is well described in the engineering literature [37,40] but has seldom been studied for aquatic animals [45]. The high-fidelity CFD simulations presented here demonstrate that drag in aquatic animals is also affected by localized morphological characteristics, as well as by the overall FR.

This study shows that the transition from narrow- to deep-bodied forms that occurred during the first 25 Myr of ichthyosaur evolution [7,21] is associated with a distinctive hydrodynamic signature that is measurable at a constant length. However, we conclude that these deep bodies were not selected for drag reduction because comparisons based on equal mass show no differences in the energy cost of steady swimming. This raises the question of what drove the change in body plan? One answer is that morphology changed just as a result of the shift in swimming mode. It is well known that shape is correlated with swimming style [7], with carangiform swimmers having deep and rigid bodies which can accommodate powerful muscles in a more efficient configuration to operate the caudal fin [6,46]. Body shape might also be linked to thermal regulation. If ichthyosaurs had acquired a raised metabolism and even thermoregulation during their evolution [47–49], then a body form with low surface area relative to volume would provide an advantage in maintaining a constant internal temperature. Regardless of the factors driving this body transformation, it occurred without bringing about a substantial reduction in drag.

The combined effect of body size and swimming mode caused a great reduction in the cost of steady locomotion during ichthyosaur evolution, mainly driven by the impact of size on drag. Propulsive efficiency depends on the velocity and kinematics, as well as the shape and flexibility of the propulsor. However, it is generally accepted that for steady swimming in the inertial regime, carangiform swimming is more efficient than anguilliform swimming [9,32,33]. Thus, assuming that Jurassic and later ichthyosaurs were carangiform/thunniform swimmers, while Triassic ones were closer to the anguilliform end of the axial undulatory spectrum [6–8], this shift in swimming mode would have reduced their energetic cost of steady locomotion by up to 50% (figure 4*a*,*b*), an effect potentiated and sometimes overridden by body size (figure 4*c*,*d*). This contribution of body size to swimming performance in ichthyosaurs has never been assessed. At life size, the small ichthyosaurs have the highest relative COT_net_ for a given speed. It is, however, unlikely that such small ichthyosaurs could sustain a speed of 1 m s^−1^ for a long time. Note that our inferences do not refer to the optimal COT_net_ (i.e. COT for optimum cruising speed, *u*_opt_). Based on living animals, it is more probable that the small forms had *u*_opt_ below 1 m s^−1^ and that medium- to large-sized ichthyosaurs had *u*_opt_ above 1 m s^−1^ [9]. Although some methods exist for inferring *u*_opt_ in fossil animals [6,11], these require a drag coefficient estimate, which conflicts with the fact that *C*_d_ (a speed-dependent value) cannot be assigned beforehand, and so we do not use these here. We instead compare COT_net_ for a standard speed and deduce that low values will provide advantages in performance, such as affording higher sustained speeds or increasing the swimming range, desirable characteristics for sustained swimmers [38]. As shown by this simple model, although moving a small body requires lower amounts of drag power (the product of total drag and velocity), which might be advantageous when available energy is limiting, if a bigger size can be afforded, this is better in terms of the energy costs per unit of mass. However, ichthyosaurs did not grow big indefinitely through their evolution (electronic supplementary material, figure S6). Body size increased rapidly during the Middle–Late Triassic [18,50], which saw the emergence of a family of giant ichthyosaurs, the shastasaurids, including forms longer than 15 m (electronic supplementary material, figure S6). After the extinction of these extremely large forms at the end of the Triassic, body size remained relatively stable and never reached these extremes again. This suggests that in spite of the selective advantage of large size in terms of the drag cost of locomotion, there are additional constraints on body size in aquatic animals, possibly related to basal metabolism, thermal exchange, feeding efficiency or food availability [51].

The marked decrease in the net cost of steady swimming that we report herein is associated with the great diversification of body size that also took place during the first 25 Myr of ichthyosaur evolution, pointing to the Early and Middle Triassic as times of rapid adaptation that saw the evolution of forms suited to a broad range of ecologies [5,52]. Our results also reveal a pattern in the energy requirements of ichthyosaur locomotion more complex than previously thought, which can be linked to their ecological adaptations. In general, Early Triassic ichthyosaurs had relatively large costs of steady locomotion owing to their small sizes and anguilliform swimming modes, although they might have been more efficient at accelerating and manoeuvring [33], making them well suited to living in near shore habitats and moving at low speeds, with no need for sustained swimming. Some Early Triassic forms, however, like *Utatsusaurus*, might have already been adapted to swimming longer distances or at higher speeds thanks at least in part to their larger sizes, in spite of being narrow-bodied anguilliform swimmers. This is in line with histological evidence that reveals a cancellous bone structure in *Utatsusaurus*, suggesting an open ocean lifestyle [53]. In fact, our results suggest that, as a lineage, ichthyosaurs were energetically well suited for life in the open ocean well before the end of the Triassic.

Here, we present to our knowledge, the first CFD-based, quantitative analysis of the drag of ichthyosaurs, based on accurate three-dimensional reconstructions of a wide array of taxa, representative of their phylogeny, morphotype disparity and body size. Our results show that from very early in their evolution, ichthyosaurs had body designs that maximized the volume for minimum drag independently of their FR, comparable to what is observed in modern cetaceans, and that further changes in the body proportions happened without measurable impact on drag. Ichthyosaurs experienced a marked reduction in the cost of steady locomotion throughout their evolution, driven mainly by body size and swimming mode.

## Supplementary Material

Supplementary methods, figures and tables

## Supplementary Material

Drag forces and coefficients from the CFD simulations

## Supplementary Material

Net cost of locomotion calculations

## Supplementary Material

Ichthyosaur body sizes

## Data accessibility

All the data relevant to this study can be found in the electronic supplementary material and downloaded from the Dryad Digital Repository: https://doi.org/10.5061/dryad.n222q81 [24].
