# Supplementary material for: Effects of body plan evolution on the hydrodynamic drag and energy requirements of swimming in ichthyosaurs
Source: Proc Biol Sci. 2019 Mar 6;286(1898):20182786. doi: 10.1098/rspb.2018.2786 (PMC6458325; doi:10.1098/rspb.2018.2786)
Supplement: Supplementary methods, figures and tables [file rspb20182786supp1.pdf]

SUPLEMENTARY INFORMATION

Effects of body plan evolution on the hydrodynamic drag and energy requirements of swimming in ichthyosaurs

Susana Gutarra[1], Benjamin C. Moon[1], Imran A. Rahman[2], Colin Palmer[1], Stephan Lautenschlager[3], Alison J. Brimacombe[1], & Michael J. Benton[1]

[1]*School of Earth Sciences, University of Bristol, Life Sciences Building, 24 Tyndall Avenue, Bristol, BS8 1TQ, United Kingdom.*

[2]*Oxford University Museum of Natural History, Parks Road, Oxford, OX1 3PW, United Kingdom.*

[3]*School of Geography, Earth and Environmental Sciences, University of Birmingham, Birmingham, B15 2TT, United Kingdom.*

Table of contents

1. **Supplementary methods** **2**
2. **Supplementary figures** **7**
3. **Supplementary tables** **14**
4. **Supplementary references** **18**

1. Supplementary methods

1.1. Ichthyosaur specimens for the creation of 3D models

We use the common term “ichthyosaur” in a broad sense to refer to all Ichthyosauriformes, the group encompassing Ichthyosauria and their closest Ichthyosauromorpha relatives [1–3]. The following ichthyosaur specimens were selected based on their excellent preservation and completeness, necessary for the creation of the three-dimensional virtual models: (i) *Cartorhynchus lenticarpus* (AGB6257 [1]) and (ii) *Chaohusaurus geishanensis* (IVPP V4001 [4]; AGM I-1, CHS-5 [5]), two basal ichthyosauriforms from the Early Triassic of China; (iii) *Utatsusaurus hataii* (UHR 30691, NSM-VP-20028 [6]), a basal ichthyopterygian from the Early Triassic of Japan; (iv) *Mixosaurus cornalianus* (MCSNM 14624; [7]), a basal ichthyosaurian from the Middle Triassic of Switzerland; (v) *Shonisaurus popularis* (specimens [8]; reconstruction[9]) and (vi) *Guizhouichthyosaurus tangae* (IVPP V11853 [10]), two shastasaurid ichthyosaurs from the Late Triassic of North America and China, respectively; (vii) *Temnodontosaurus platyodon* (NHMUK 2003 [11]; pers. obs. by A.J.B.), a neoichthyosaurian from the Lower Jurassic of Europe; (viii) *Stenopterygius quadriscissus* (NHMUK R4086; pers. obs. by A.J.B.), a thunnosaurian ichthyosaur from the Lower Jurassic of Europe; and (ix) *Ophthalmosaurus icenicus* (NHMUK PV R3702, R3898, R4124; pers. obs. by A.J.B. and B.C.M., [12]), a derived thunnosaurian ichthyosaur from the Middle–Upper Jurassic of Eurasia.

Original specimens of *Ophthalmosaurus*, *Stenopterygius* and *Temnodontosaurus* were studied and photographed in different orientations. Additional measurements were made from skeletal elements relevant for the three-dimensional model (i.e. the skull, the extent of the ribs and the dimensions of the limbs). Data for *Cartorhynchus*, *Chaohusaurus*, *Utatsusaurus*, *Mixosaurus*, *Shonisaurus* and *Guizhouichthyosaurus* were obtained from published skeletal reconstructions and soft-tissue outlines [5,9,10,13,14].

1.2. 3-D reconstruction of fossil ichthyosaurs and a bottlenose dolphin

For each ichthyosaur species, a three-dimensional model was created using the 3-D computer graphics software Rhinoceros, v. 5.5.3 (Figure 1; Figure S1A). Photographs and published skeletal reconstructions in lateral and dorsal views and, where possible, coronal cross-sections, were imported into Rhinoceros as background images. For model creation, a non-uniform rational basis spline (NURBS) modelling approach was employed; this technique is well suited for the representation of organic shapes through deformable, smooth, mathematically-defined surfaces [15]. A curve that defined the body outline of the ichthyosaur was created and then revolved around the longitudinal axis to generate the trunk. In most cases, the lateral view was used to create the revolved body, as this orientation provided the most information from the preserved soft-body outlines or reconstructions. Subsequently, the models were adjusted in dorsal view. The width of the ribs in most cases is not preserved in the fossils. For this reason,

a standard width based on a three-dimensionally mounted skeleton, that of *Ophthalmosaurus icenicus* [13], was used for all specimens (Figure S1A). The fore- and hind-limbs were constructed using a sweep-two rail of the fin profile and a cross-section. In the absence of soft tissue outlines, an extra margin equivalent to that observed in specimens with preserved fin outlines was added to the edges of the bones to create the planform shape. In all cases, the cross-section of the fins was based on that of a modern dolphin [16]. The thickness of the pectoral and caudal fins is determined from the dorsoventral width of the limb bones. This information is unfortunately not available for most specimens, due to the lack of three-dimensional preservation. *Ophthalmosaurus* is an exception, as a three-dimensionally preserved specimen, from which the limb bone thickness could be measured directly, is known [17]. Incorporating this information into the model resulted in relatively thicker fins compared to the dolphin model (about 1.5 times thicker), reflecting the robustness of *Ophthalmosaurus* limb bones. All other ichthyosaur models have slightly variable flipper thicknesses that are intermediate between the *Tursiops* and *Ophthalmosarus* models, as a result of the cross-section being adapted to different flipper contours. This source of variability has been addressed by sensitivity tests (see section 1.5). For consistency, the limbs were orientated at an angle of 45° relative to the sagittal plane in all models, and they were feathered. The animals were designed as bilaterally symmetrical geometries, applying the 'mirroring' tool to the body and limbs. The limbs and body were joined by a Boolean union, and a fillet was created to smooth the transition between them. The dorsal fin and caudal fluke were created using the same technique, and were also based on a dolphin's dorsal fin and fluke cross-sections [16]. The digital model of a bottlenose dolphin, *Tursiops truncatus*, a modern functional analogue of ichthyosaurs, was constructed from lateral and dorsal views of anatomically accurate life reconstructions [18] and photographs, which provided information on the depth and width of the body, respectively, as well as the outline profiles of the appendages.

For use in the CFD simulations, the models were exported as STP files. The only known specimen of *Cartorhynchus* has an incomplete tail and, for this reason, two digital versions were prepared for this taxon (Table S1): a 'short tail' model with the tail length of a basal ichthyopterygian and a 'long tail' model with a tail proportion similar to its closest relative, the nasorostran *Sclerocormus parviceps*, based on data published elsewhere [2]. The results for *Cartorhynchus* have been averaged from both versions (Data S1 and Data S2), but the graphical representations in Figures 1–4 and Figure S3 only display the 'long tail' model.

1.3. Computational fluid dynamics

(a) Mesh characteristics

The flow domain of each model was divided into discrete mesh elements with ANSYS-Meshing, following standard mesh criteria for the simulation of external aerodynamic flows. Tetrahedral elements were used in the free flow region (i.e. interior of the virtual cylinder) and the symmetry boundaries (i.e. the walls of

the virtual cylinder). An inflation layer with 20–25 layers of prismatic elements was created at the no-slip boundaries (i.e. the walls of the model) to model the boundary layer region. The number of inflation layers was determined as well through mesh sensitivity tests. Finally, an area of extra refinement was added around the wake (Figure S1D and S1E). This refinement area consisted of a box with a height of 2× the maximum diameter of the model and a length of 1.5× the length of the model, dimensions that were determined empirically as the minimum size that did not affect the results. The number and minimum/maximum size of the mesh elements used in the CFD simulations varied depending on the complexity and size of the model. To test whether the results were independent of mesh size, we carried out mesh independence tests for each model with no fins at a Reynolds number of 10^7. The mesh was refined, producing increasingly large numbers of mesh elements for the simulations. A solution was considered mesh independent if the converged value for drag did not change by more than 1% between a mesh and the next coarsest one. This was then selected as the mesh of choice for the rest of the analysis.

(b) Flow regime and turbulence model

The flow regime for the simulation experiments is determined by the magnitude of the Reynolds number (*Re,* also referred to as Re_L herein), defined by the equation:

$$Re = \frac{\rho \, V \, L}{\mu}$$

Where ρ is the density (998.2 kg m^{-3}) and μ is the dynamic viscosity (1.002 10^{-3} Pa s) of water at 20°C, *u* is the free-stream velocity (m s^{-1}) and *L* is the body length (m). The Reynolds number defines the balance of viscous and inertial forces that act on the liquid particles [19] and determines the properties of the boundary layer (i.e. the thin layer of liquid that extends from the surface of the object, where velocity is zero, to the area of free stream velocity). Below a Reynolds number of 10^3, the flow regime is laminar, with liquid particles that move describing smooth and orderly streamlines. A fully turbulent regime occurs at Reynolds numbers above 10^4, with particles that move chaotically and form vortices within the boundary layer. Transitional flow regimes can occur at Reynolds numbers between these two magnitudes, although these boundaries are not rigid and might vary with the geometry of the objects and surface roughness.

Based on the dimensions of the specimens under study and the speed at which modern animals of similar sizes move, we chose to use the fully turbulent, shear stress transport (SST) to solve the Reynolds Averaged Navier Stokes (RANS) equations. The SST turbulence model combines the *k*-*ε* model in the free stream and the *k*-*ω* model near the walls [20], which results in an improved performance under adverse pressure gradients and hence in a more accurate prediction of flow separation [21], likely to develop at the rear part of the models. The characteristics of the mesh at the boundary layer region were

adjusted to for the optimal performance of this CFD protocol. The *y*+ values (dimensionless distance to the wall) for the simulations were in all cases <5, to ensure that the first node of the inflation layer felt within the viscous sublayer, as required for turbulence models that don't use wall functions.

1.4. Validation of the CFD protocol

Prior to the analyses with the ichthyosaur models, we conducted a validation of the CFD parameters. This consisted of fine-tuning the program settings to replicate the results from real water tank experiments carried out on a set of streamlined shapes. The Series 58 bodies of revolution [22] (i.e. rotationally symmetrical) were selected for the validation experiments. These slender bodies represent a good analogue for swimming vertebrates like ichthyosaurs and their hydrodynamic properties at Reynolds numbers within the range of our analyses have been studied extensively during the last century [23]. Three rotational bodies of this series were selected, the 4154, 4157 and 4159, with fineness ratios of 4, 7 and 10, respectively (Figure S2), to cover a similar range to that of our ichthyosaur models (Table S1). They were modelled in Rhinoceros (v. 5.5.3) based on their mathematical polynomial curves [60], and water flows were simulated in ANSYS at various speeds corresponding to Reynolds numbers of 4×10^6 to 2×10^7. Our CFD results were compared to the experimental fully-turbulent drag coefficients, obtained from experiments in which the turbulence has been forced by placing sand-strips on the nose of the objects (Data S1).

1.5. CFD Sensitivity tests for the ichthyosaur models

The drag of the ichthyosaurs was evaluated at zero (or very close to zero) lift, so that the contribution from the induced drag was negligible. The models were initially tested at their original orientation and, in cases where a large amount of lift was produced, small corrections to the angle of attack were made to ensure that the induced drag contributed to less than 1% of the total drag (Figure S4A and S4B). Sensitivity tests were conducted to account for the effect of modelling uncertainties on the results. A series of simulations run with versions of the *Ophthalmosaurus* model of varying lateral widths showed that there is a large margin of variation within which the drag coefficient is not substantially affected (Figure S4C). Additionally, two parameters of the flippers were evaluated. Versions of *Ophthalmosaurus* with different overall thicknesses of the pectoral and pelvic flippers, as well as various thicknesses of the fin insertions were tested to ensure that differences between ichthyosaurs would not penalize any of them with excessively high total pressure drag or interference drag. These tests showed that there is a safe range of variation in which the thickness at the base of the fins has a negligible effect on the total drag (less than 2% for most of the range examined; Figure S4D, solid line) and overall thickness has only a small impact (≤3% increase for each 20% change; Figure S4D, dotted line). However small this effect, we acknowledge that variation in the cross-section profile, width and planform shape of the flippers can be a source of uncertainty, which confirms the need to apply a standard criterion when modelling soft tissues

of aquatic animals (in our case, a uniform cross-section, as used by previous studies[24]) and discourages us from ranking the taxa studied herein based on the differences in the drag of their fins.

1.6. Statistical analysis

All statistical analyses were carried out in R [25]. To compare the mean values of the drag coefficient and relative drag per unit volume between ichthyosaur grades, we used a two-sample *t*-test. We assessed normal distribution and homoscedasticity of each group's data with the Shapiro-Wilk's test and the Bartlett test respectively. Only the dataset corresponding to the drag coefficients of the basal grade ichthyosaurs was found to deviate from normality; in this case, we used the Fligner-Killeen test to assess variance homogeneity and the non-parametric Mann-Whitney U test for group comparisons. Correlation between the mean drag coefficient and the mean occurrence age of ichthyosaurs was tested using the non-parametric Kendall's rank correlation. This test was chosen because it is sensitive to non-linear relationships and because of the non-normal distribution of the age occurrences. Kendall's tau (τ) indicates the strength of the correlation.

1.7. Body size of ichthyosaurs

Values of total body length (i.e. distance from the tip or the rostrum to the tip of the tail) for a wide selection of ichthyosaurs, including the nine taxa under study, were compiled from literature estimations or measured in ImageJ from scaled photographs of complete specimens or reconstructions (Data S3). The mean body length, calculated per genus, was plotted against the average occurrence age (Figure S6). The mean, minimum and maximum body length values for the nine taxa used in the present study were used to perform CFD simulations at a velocity of 1 m s^{-1} to calculate the COT_{net} at life-size dimensions (Data S2 and Figure 4*c* and 4*d*).

1.8. Institutional abbreviations

AGM, Anhui Geological Museum, Anhui, China; IVPP, Institute of Vertebrate Paleontology and Paleoanthropology, Beijing, China; MCSNM, Museo Civico di Storia Naturale, Milan, Italy; NHMUK, Natural History Museum, London, UK; NSM, National Science Museum, Tokyo, Japan; UHR, Hokkaido University, Sapporo, Japan.

2. Supplementary figures

A

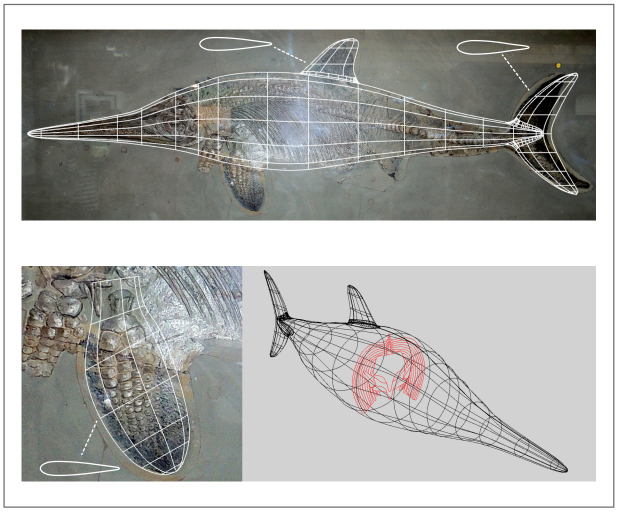

B

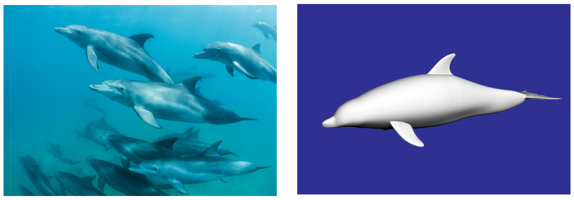

	Measurements from live dolphin (Fish, 1988)	Measurements from 3D model based on 2D image (Rhinoceros)
L (m)	2.51 - 2.70	2.51 - 2.70
D (m)	0.48 - 0.58	0.51 - 0.55
S (m^2)	2.45 - 2.99	2.56 - 2.96
V (m^3)	NA	0.18 - 0.22
m (kg)	192.8 - 263.1	187.9 - 233.9

C

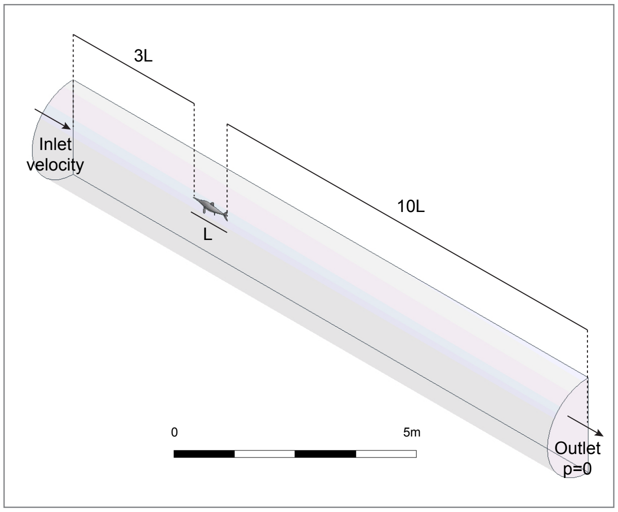

D

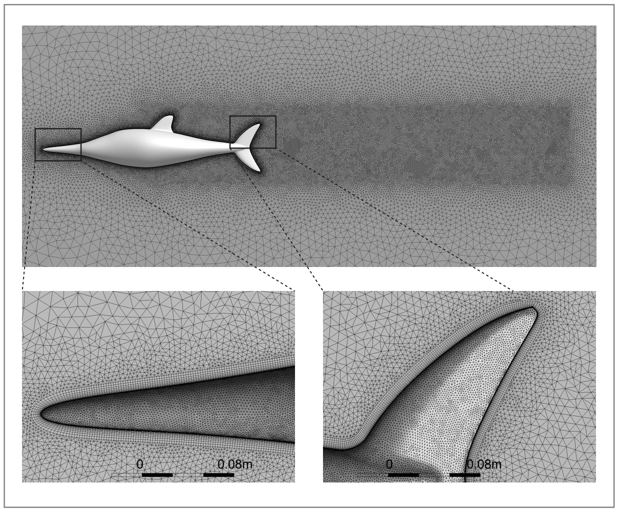

E

CFD Parameters	Settings
Mesh type	Tetrahedral with refinement area aound wake plus 25 prismatic inflation layers
Nº of mesh elements	12 - 20 M
y+	<5
Turbulence model	k-omega shear stress transport (SST k-ω)
Convergence criteria	Stable numerical solution for drag<br>RMS residual $<10^{-4}$<br>Mass flow rate imbalance <1%

Figure S1. Methodology workflow. (A) Modelling approach for the body profile (upper and lower-right panel) and fins (lower-left panel). The outline profiles used for the cross-sections of the dorsal, caudal and pectoral fins [16] are displayed. (B) Comparison between estimated parameters extracted from the digital reconstruction and living specimens of *Tursiops truncatus* [26]. Measurements correspond to: total body length, L; maximum body diameter, D; wetted surface area, S; body volume, V; and mass, m. The mass was calculated by multiplying the volume, V, by the density of sea water (1.025 kg m^{-3}). (C) Flow domain around an ichthyosaur model created in ANSYS DesignModeler, showing the dimensions relative to the total length (L) of the model. (D) Mesh created with ANSYS Meshing, showing the tetrahedral elements and the area of refinement around the wake (upper panel); details of the boundary layer at the tip of the head (lower-left) and read end (lower-right) of the model. (E) Computational parameters of the CFD analyses.

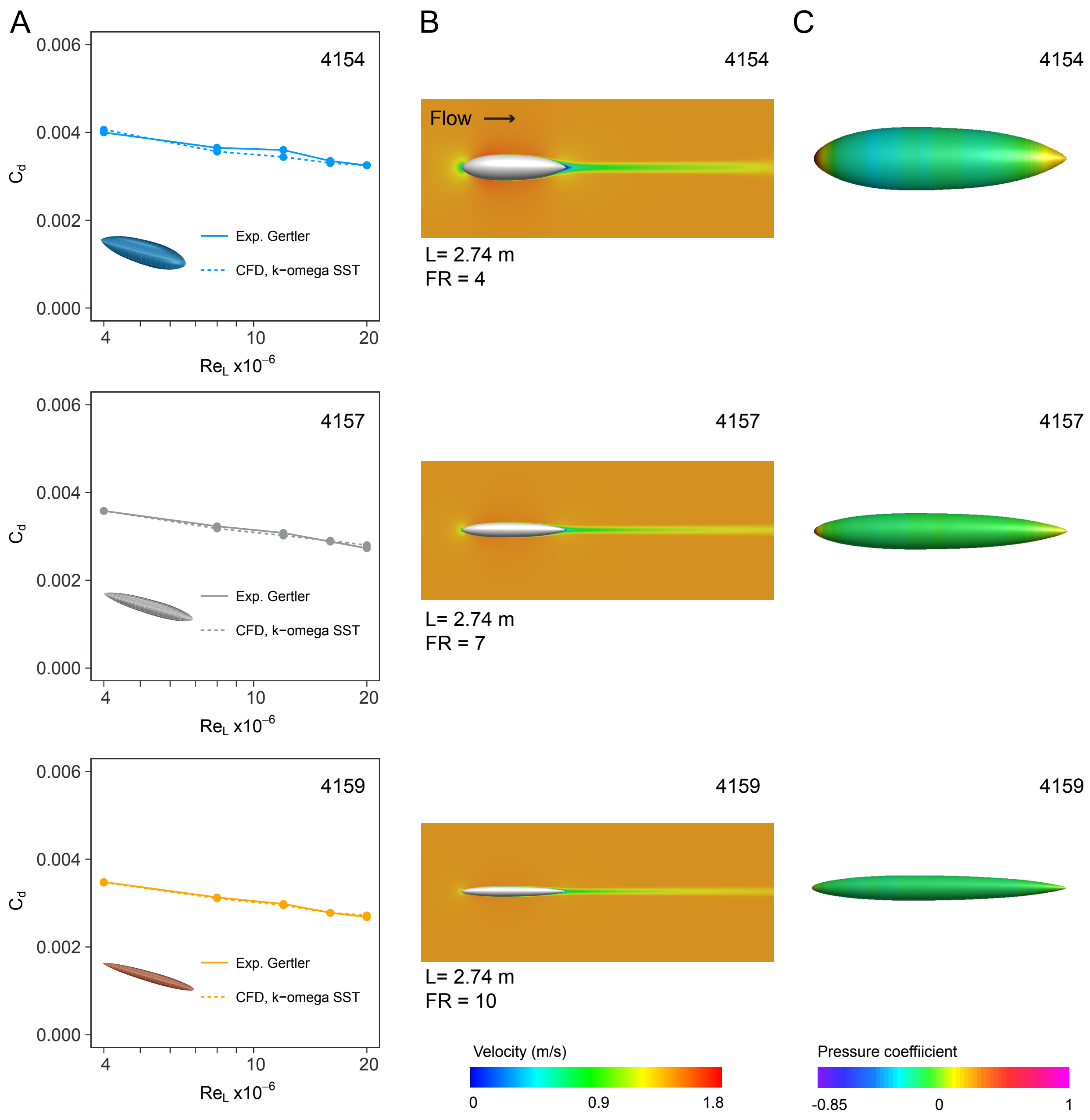

Figure S2. Validation of CFD method using standard bodies of revolution. (A) Experimental (solid lines) and computed CFD (dashed lines) drag coefficients of three rotational slender bodies of the Series 58 [23] at zero-lift, over a range of Reynolds number from 4×10^6 to 2×10^7. (B) Two-dimensional velocity plots at a $Re = 4 \times 10^6$. The total length, L, and fineness ratio, FR (length divided by the maximum diameter) are displayed. (C) Pressure coefficient plots of the three rotational bodies at a $Re = 4 \times 10^6$.

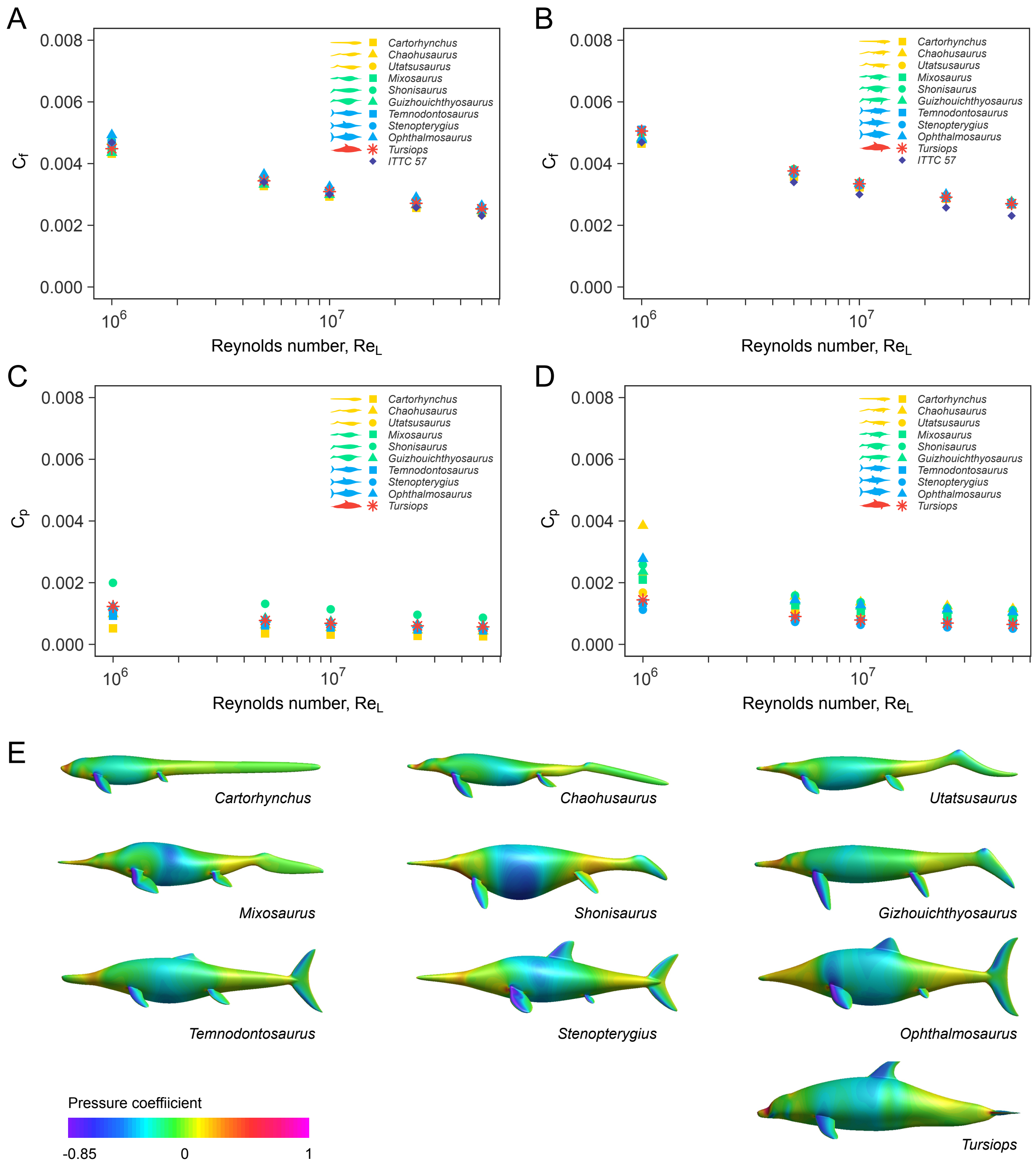

Figure S3. Friction and pressure drag coefficient profiles for ichthyosaurs with and without limbs. (A-B) CFD-computed skin friction drag coefficients of nine ichthyosaurs and a bottlenose dolphin without (A) and with (B) limbs at Reynolds number from 10^6 to 5×10^7. (C-D) CFD-computed pressure drag coefficients of nine ichthyosaurs and a bottlenose dolphin without (C) and with (D) limbs at Reynolds number from 10^6 to 5×10^7. Ichthyosaurs from the ‘basal grade’ are highlighted in yellow, the ‘intermediate grade’ in green, and the ‘fish-shaped ichthyosaurs’ in blue. The bottlenose dolphin *Tursiops* is highlighted in red. (E) Three-dimensional plots showing the pressure coefficient distribution for a Reynolds number of 5×10^6 (inlet velocity of 5 m s^{-1}).

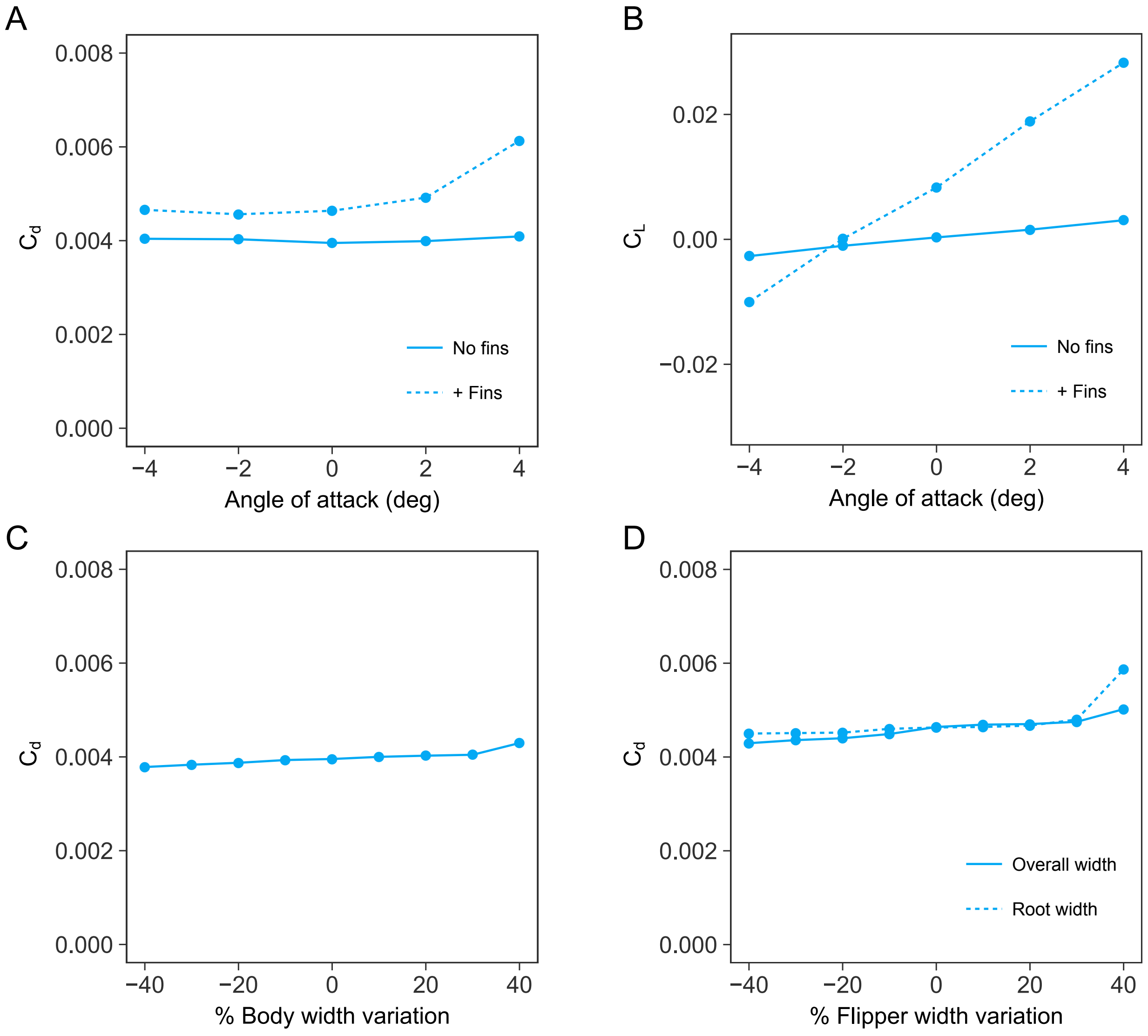

Figure S4. Sensitivity of the results to variations in orientation and modelling uncertainties. (A-B) Effect of the angle of attack, measured in degrees, on the total drag coefficient (A) and lift coefficient (B) in the *Ophthalmosaurus* model without limbs (solid line) and with limbs (dashed lines). All the results in Figure 2 come from simulations with a lift coefficient close to 0. (C) Effect of the body lateral width on the drag coefficient of *Ophthalmosaurus* with no limbs. (D) Effect of the overall thickness of the limbs (solid line) and the thickness of the limb insertions (dashed lines) in the full model of *Ophthalmosaurus*. All simulations correspond to *Re* = 10^7 (velocity inlet = 10 m s^{-1}).

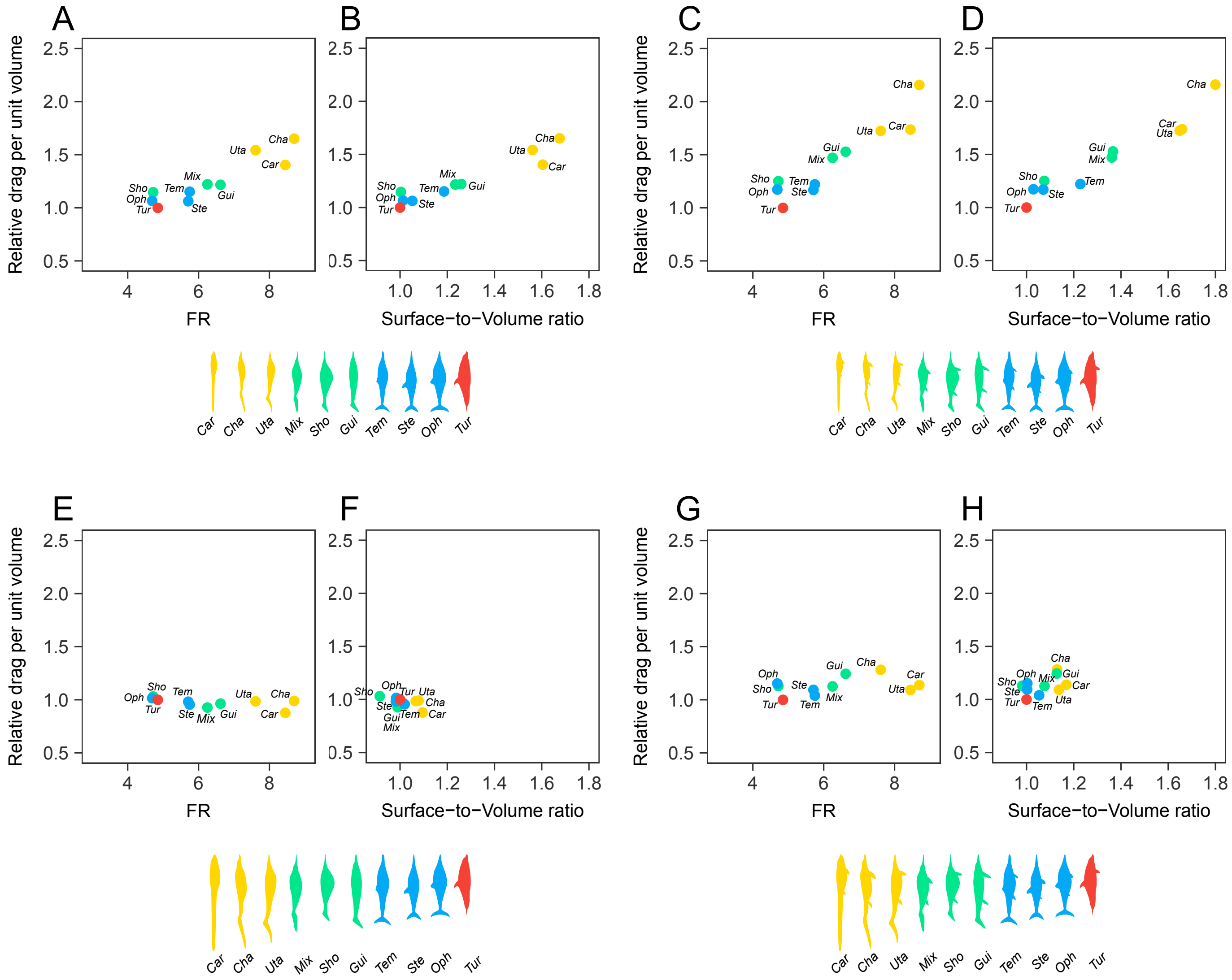

Figure S5. Relative drag per unit of volume versus the fineness ratio (FR) and the surface area-to-volume ratio. (A to D) Relative values of drag per unit of volume for ichthyosaurs scaled to the same length and tested at the same speed, with (A and B) and without (C and D) limbs, plotted against the fineness ratio, FR (A and C) and the surface area-to-volume ratio (B and D). (E to H) Relative values of drag per unit of volume for ichthyosaurs scaled to the same volume and tested at the same speed, with (E and F) and without (G and H) limbs, plotted against the fineness ratio, FR (E and G) and the Surface-to-volume ratio (F and H). Ichthyosaurs from the 'basal grade' are highlighted in yellow, the 'intermediate grade' in green, and the 'fish-shaped ichthyosaurs' in blue. The bottlenose dolphin *Tursiops* is highlighted in red.

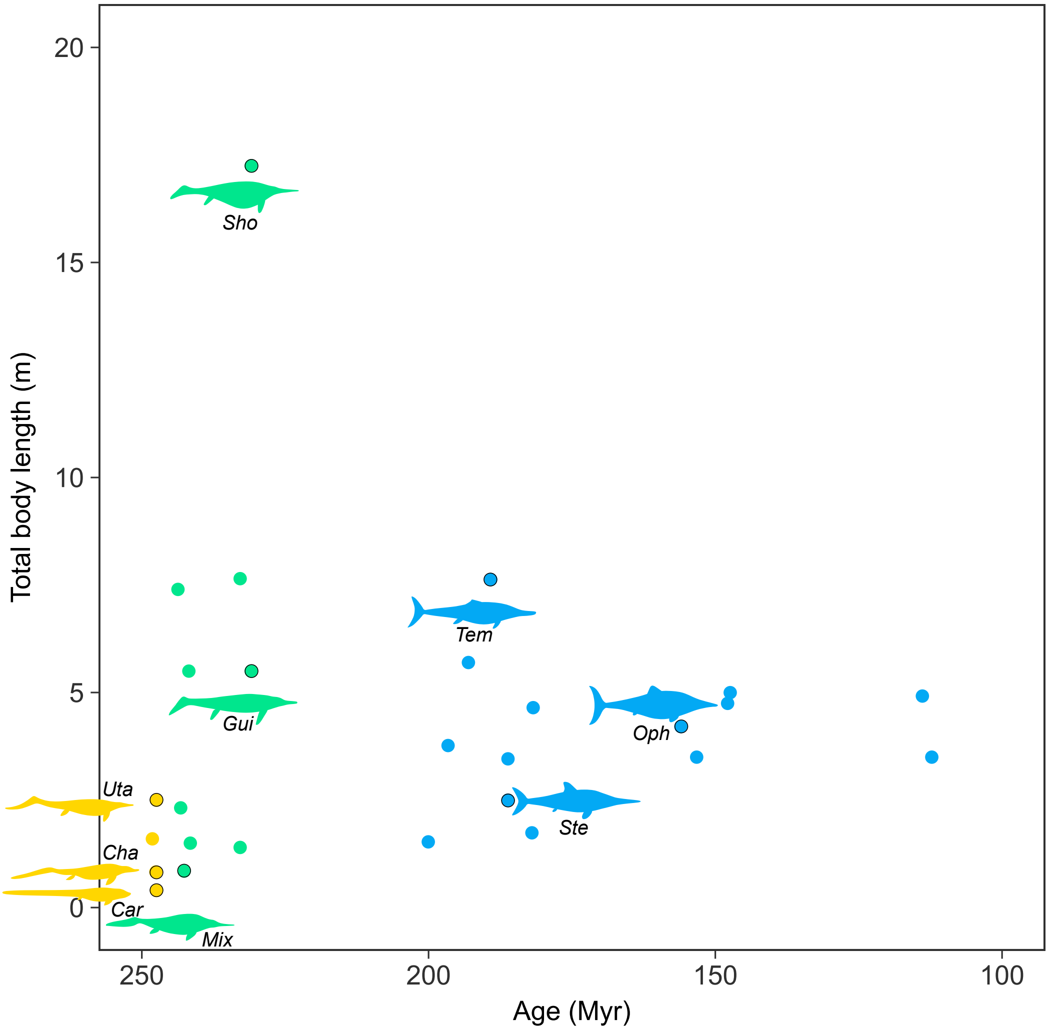

Figure S6. Body length of ichthyosaurs. Measurements of total body length (distance from the tip of the rostrum to the tip of the tail) were collected from the literature for a wide array of ichthyosaur genera (Data S3). Average values per genus were calculated and plotted against the mean occurrence in millions of years. Ichthyosaurs from the 'basal grade' are highlighted in yellow, the 'intermediate grade' in green, and the 'fish-shaped ichthyosaurs' in blue.

3. Supplementary tables

	Taxon	L (m)	D (m)	FR	S (m^2)	V (m^3)	% Limb SA / S
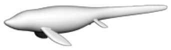	*Cartorhynchus* short tail	1	0.129	7.72	0.23	0.0044	13.66
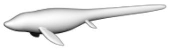	*Cartorhynchus* long tail	1	0.109	9.17	0.19	0.0029	11.4
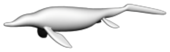	*Chaohusaurus*	1	0.110	8.70	0.200	0.0032	16.2
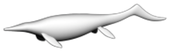	*Utatsusaurus*	1	0.131	7.61	0.216	0.0037	13.0
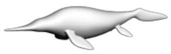	*Mixosaurus*	1	0.150	6.25	0.274	0.0057	16.4
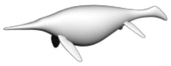	*Shonisaurus*	1	0.210	4.72	0.334	0.0088	14.1
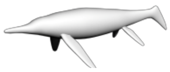	*Guizhouichthyosaurus*	1	0.151	6.62	0.313	0.0065	18.3
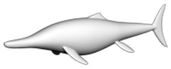	*Temnodontosaurus*	1	0.174	5.75	0.316	0.0073	9.8
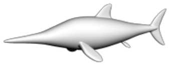	*Stenopterygius*	1	0.180	5.71	0.358	0.0095	10.0
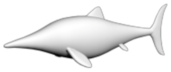	*Ophthalmosaurus*	1	0.213	4.69	0.387	0.0107	9.7
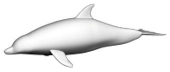	*Tursiops*	1	0.206	4.85	0.407	0.0116	6.7

Table S1. Geometric parameters of the reconstructed taxa scaled to the same total length. Table showing dimensions of the nine ichthyosaurs and a bottlenose dolphin models scaled to a full length of 1 m, measured in Rhinoceros (v. 5.5.3). L is the total body length, from the tip of the rostrum to the tip of the tail; D is the maximum body perpendicular to the longitudinal axis; FR is the fineness ratio calculated as L divided by D; S is the total (or wetted) surface area; V is the total volume; % Limb SA / S is the percentage of surface area of limbs in relation to the total body surface.

Body length = 1 m

	No limbs					+Limbs				
		u = 1 m/s		u = 2 m/s			u = 1 m/s		u = 2 m/s	
Taxon	V (m^3)	Drag (N)	D/V	Drag (N)	D/V	V (m^3)	Drag (N)	D/V	Drag (N)	D/V
Cartorhynchus	0.0036	0.46	131.0	1.60	459.6	0.0037	0.72	203.7	2.39	673.0
Chaohusaurus	0.0031	0.48	157.1	1.68	547.0	0.0032	0.87	273.7	2.59	816.7
Utatsusaurus	0.0037	0.53	145.8	1.87	507.1	0.0037	0.72	192.6	2.47	664.2
Mixosaurus	0.0056	0.64	114.3	2.23	398.7	0.0057	0.98	170.1	3.17	553.3
Shonisaurus	0.0087	0.97	111.6	3.30	379.7	0.0088	1.28	144.8	4.14	468.5
Guizhouichthyosaurus	0.0064	0.73	113.6	2.55	398.0	0.0065	1.12	171.6	3.80	581.9
Temnodontosaurus	0.0073	0.79	109.4	2.74	378.7	0.0074	1.02	139.1	3.38	461.3
Stenopterygius	0.0094	0.94	100.3	3.28	350.8	0.0095	1.54	161.2	5.22	547.5
Ophthalmosaurus	0.0106	1.09	102.8	3.63	343.7	0.0107	1.46	135.9	4.72	440.4
Tursiops	0.0115	1.08	94.3	3.77	327.6	0.0116	1.32	113.7	4.30	370.5

Body length = 2 m

	No limbs							+Limbs						
		u = 1 m/s		u = 2.5 m/s		u = 5 m/s			u = 1 m/s		u = 2.5 m/s		u = 5 m/s	
Taxon	V (m^3)	Drag (N)	D/V	Drag (N)	D/V	Drag (N)	D/V	V (m^3)	Drag (N)	D/V	Drag (N)	D/V	Drag (N)	D/V
Cartorhynchus	0.028	1.60	57.4	8.57	307.2	30.62	1097.6	0.029	2.39	84.1	12.24	428,5	43.91	1537.2
Chaohusaurus	0.025	1.68	68.4	8.86	361.2	31.53	1284.8	0.025	2.59	102.1	13.25	522.7	47.70	1881.4
Utatsusaurus	0.029	1.87	63.6	9.90	337.3	35.40	1206.0	0.029	2.47	83.0	13.04	436.1	46.42	1553.2
Mixosaurus	0.045	2.23	49.8	11.89	265.9	42.60	952.5	0.046	3.17	72.5	17.20	374.8	60.16	1311.1
Shonisaurus	0.070	3.30	47.4	17.49	251.3	61.93	889.8	0.071	4.14	58.6	22.52	318.4	79.27	1120.6
Guizhouichthyosaurus	0.051	2.55	49.8	13.55	264.8	48.56	948.7	0.052	3.80	72.7	20.00	382.6	71.53	1368.3
Temnodontosaurus	0.058	2.74	47.2	14.60	251.4	52.15	898.2	0.059	3.38	59.4	18.19	310.1	64.49	1099.4
Stenopterygius	0.075	3.28	43.7	17.39	232.1	62.11	828.9	0.076	5.22	68.4	24.85	325.8	88.04	1154.3
Ophthalmosaurus	0.085	3.63	43.0	19.66	232.7	69.85	826.6	0.086	4.72	55.1	25.02	291.6	89.68	1045.3
Tursiops	0.092	3.77	40.9	20.02	217.5	71.69	778.8	0.093	4.30	46.3	23.67	255.2	83.93	904.8

Body length = 10 m

	No limbs							+Limbs						
		u = 1 m/s		u = 2.5 m/s		u = 5 m/s			u = 1 m/s		u = 2.5 m/s		u = 5 m/s	
Taxon	V (m^3)	Drag (N)	D/V	Drag (N)	D/V	Drag (N)	D/V	V (m^3)	Drag (N)	D/V	Drag (N)	D/V	Drag (N)	D/V
Cartorhynchus	3.56	30.62	8.78	167.7	48.1	626.5	179.6	3.64	43.91	12.3	241.5	67.6	901.0	252.4
Chaohusaurus	3.07	31.53	10.28	171.4	55.9	639.6	208.5	3.17	47.70	15.1	262.8	82.9	976.2	308.0
Utatsusaurus	3.67	35.40	9.65	192.3	52.4	717.3	195.5	3.74	46.42	12.4	252.9	67.7	946.3	252.6
Mixosaurus	5.59	42.60	7.62	238.0	42.6	866.2	154.9	5.74	60.16	10.5	329.5	57.4	1231.7	214.4
Shonisaurus	8.72	61.93	7.10	336.1	38.5	1244.2	142.7	8.84	79.27	9.0	431.5	48.8	1612.7	182.4
Guizhouichthyosaurus	6.40	48.56	7.59	268.8	42.0	998.4	156.1	6.53	71.53	10.9	397.4	60.8	1471.9	225.2
Temnodontosaurus	7.26	52.15	7.19	285.0	39.3	1064.6	146.7	7.33	64.49	8.8	349.8	47.7	1308.6	178.5
Stenopterygius	9.37	62.11	6.63	338.9	36.2	1264.4	135.0	9.53	88.04	9.2	485.8	51.0	1819.4	190.8
Ophthalmosaurus	10.56	69.85	6.61	388.9	36.8	1415.4	134.0	10.72	89.68	8.4	500.5	46.7	1834.5	171.1
Tursiops	11.51	71.69	6.23	393.3	34.2	1472.8	128.0	11.59	83.93	7.2	456.6	39.4	1698.2	146.5

Table S2. Calculations of drag per unit volume for models scaled to the same total length. The total drag force, D has been divided by the volume, V to obtain the drag per unit of volume, D/V, in N m^{-3}, for various combinations of inlet velocity, u, and body length, L. The results for *Cartorhynchus* are the average from two model versions with different tail lengths.

Body volume (V) = 0.1 m^3

Taxon	No limbs			+Limbs		
	u = 1 m/s			u = 1 m/s		
	L (m)	Drag (N)	D/V	L (m)	Drag (N)	Drag (N) D/V
Cartorhynchus	3.04	3.48	34.78	3.05	4.90	2.39
Chaohusaurus	3.19	3.91	39.12	3.16	5.79	2.59
Utatsusaurus	3.01	3.92	39.24	2.99	5.11	2.47
Mixosaurus	2.61	3.73	37.27	2.59	5.06	3.17
Shonisaurus	2.25	4.11	41.12	2.24	5.08	4.14
Guizhouichthyosaurus	2.5	3.82	38.19	2.48	5.59	3.80
Temnodontosaurus	2.39	3.79	37.91	2.39	4.67	3.38
Stenopterygius	2.20	3.89	38.95	2.19	4.91	5.22
Ophthalmosaurus	2.11	4.04	40.36	2.11	5.20	4.72
Tursiops	2.05	3.98	39.77	2.05	4.49	4.30

Body volume (V) = 0.5 m^3

Taxon	No limbs			+Limbs		
	u = 1 m/s			u = 1 m/s		
	L (m)	Drag (N)	D/V	L (m)	Drag (N)	Drag (N) D/V
Cartorhynchus	5.19	9.28	18.56	5.21	13.06	26.12
Chaohusaurus	5.46	10.40	20.81	5.40	15,24	30.48
Utatsusaurus	5.14	10.45	20.90	5.12	13.61	27.22
Mixosaurus	4.47	9.67	19.34	4.43	13.44	26.88
Shonisaurus	3.85	10.84	21.68	3.84	13.47	26.94
Guizhouichthyosaurus	4.27	10.19	20.38	4.25	14.86	29.71
Temnodontosaurus	4.09	10.09	20.19	4.09	12.42	24.85
Stenopterygius	3.76	10.38	20.76	3.74	13.07	26.12
Ophthalmosaurus	3.62	10.71	21.42	3.60	13.71	27.42
Tursiops	3.51	10.53	21.07	3.51	11.93	23.85

Table S3. Calculations of drag per unit volume for models scaled to the same total volume. The total drag force, D has been divided by the volume, V to obtain the drag per unit of volume, D/V, in N m^{-3}, for various combinations of body volume, at the same inlet velocity of 1 m s^{-1}. The results for *Cartorhynchus* are the average from two model versions with different tail length.

Supplementary references

1. Motani R, Jiang D-Y, Chen G-B, Tintori A, Rieppel O, Ji C, Huang J-D. 2015 A basal ichthyosauriform with a short snout from the Lower Triassic of China. *Nature* **517**, 485–488. (doi:10.1038/nature13866)

2. Jiang D-Y *et al.* 2016 A large aberrant stem ichthyosauriform indicating early rise and demise of ichthyosauromorphs in the wake of the end-Permian extinction. *Sci. Rep.* **6**, 26232. (doi:10.1038/srep26232)

3. Moon BC. 2017 A new phylogeny of ichthyosaurs (Reptilia: Diapsida). *J. Syst. Palaeontol.* **17**, 129–155. (doi:10.1080/14772019.2017.1394922)

4. Motani R, Jiang D-Y, Tintori A, Rieppel O, Chen G-B, You H. 2015 Status of *Chaohusaurus chaoxianensis* (Chen, 1985). *J. Vertebr. Paleontol.* **35**, e892011. (doi:10.1080/02724634.2014.892011)

5. Motani R, Jiang D, Tintori A, Rieppel O, Chen G. 2014 Terrestrial origin of viviparity in Mesozoic marine reptiles Indicated by Early Triassic embryonic fossils. *PLoS ONE* **9**, e88640. (doi:10.1371/journal.pone.0088640)

6. Motani R. 1998 Ichthyosaurian relationships illuminated by new primitive skeletons from Japan. *Nature* **393**, 255–257. (doi:10.1038/30473)

7. Brinkmann W. 1999 *Ichthyosaurus cornalianus* Bassani, 1886 (currently *Mixosaurus cornalianus*, Reptilia, Ichthyosauria); proposed designation of a neotype. *Bull. Zool. Nomencl.* **56**, 247–249.

8. Camp CL. 1980 Large ichthyosaurs from the Upper Triassic of Nevada. *Palaeontogr. Abt. A* **170**, 139–200.

9. Kosch BF. 1990 A revision of the skeletal reconstruction of *Shonisaurus popularis* (Reptilia: Ichthyosauria). *J. Vertebr. Paleontol.* **10**, 512–514. (doi:10.1080/02724634.1990.10011833)

10. Shang Q-H, Li C. 2009 On the occurrence of the ichthyosaur *Shastasaurus* in the Guanling biota. *Vertebr. Palasiat.* **47**, 178–183.

11. McGowan C. 1974 A revision of the longipinnate ichthyosaurs of the Lower Jurassic of England, with descriptions of two new species (Reptilia: Ichthyosauria). *Life Sci. Contrib. R. Ont. Mus.* **97**, 1–37.

12. Andrews CW. 1915 I.—Note on a Mounted Skeleton of *Ophthalmosaurus icenicus*, Seeley. *Geol. Mag.* **2**, 145–146.

13. McGowan C, Motani R. 2003 *Handbook of Paleoherpetology: Ichthyopterygia*. München: F. Pfeil.

14. Motani R, Jiang D, Tintori A, Ji C, Huang J. 2017 Pre- versus post-mass extinction divergence of Mesozoic marine reptiles dictated by time-scale dependence of evolutionary rates. *Proc. R. Soc. B Biol. Sci.* **284**, 20170241. (doi:10.1098/rspb.2017.0241)

15. Hutchinson JR, Ng-Thow-Hing V, Anderson FC. 2007 A 3D interactive method for estimating body segmental parameters in animals: Application to the turning and running performance of *Tyrannosaurus rex*. *J. Theor. Biol.* **246**, 660–680. (doi:10.1016/j.jtbi.2007.01.023)

16. Fish FE. 2006 The myth and reality of Gray's paradox: implication of dolphin drag reduction for technology. *Bioinspir. Biomim.* **1**, R17–R25. (doi:10.1088/1748-3182/1/2/R01)

17. Moon BC, Kirton AM. 2016 Ichthyosaurs of the British Middle and Upper Jurassic. PART 1. *Ophthalmosaurus*. *Monogr. Palaeontogr. Soc.* **170**, 1–84.

18. Jefferson TA, Webber MA, Pitman R. 2015 *Marine mammals of the world*. 2nd edn. Academic Press.

19. Schlichting H, Gersten K. 2017 *Boundary-layer theory*. 9th edn. Berlin, Heidelberg: Springer Berlin Heidelberg. (doi:10.1007/978-3-662-52919-5)

20. Menter FR. 1994 Two-equation eddy-viscosity turbulence models for engineering applications. *AIAA J.* **32**, 1598–1605. (doi:10.2514/3.12149)

21. Phillips A, Furlong M, Turnock SR. 2007 The use of computational fluid dynamics to assess the hull resistance of concept autonomous underwater vehicles. In *Oceans 2007-Europe*, pp. 1292–1297. Richardson TX, USA: Institute of Electrical and Electronics Engineers, IEEE. (doi:10.1109/OCEANSE.2007.4302434)

22. Landweber L, Gertler M. 1950 Mathematical formulation of bodies of revolution. Report R-719. The David W Taylor Model Basin, Washington DC.

23. Gertler M. 1950 Resistance experiments on a systematic series of streamlined bodies of revolution - for application to the design of high-speed submarines. Report C-297. The David W Taylor Model Basin, Washington DC.

24. Muscutt LE, Dyke G, Weymouth GD, Naish D, Palmer C, Ganapathisubramani B. 2017 The four-flipper swimming method of plesiosaurs enabled efficient and effective locomotion. *Proc. R. Soc. B Biol. Sci.* **284**, 20170951. (doi:10.1098/rspb.2017.0951)

25. Team RC. 2016 *R: A language and environment for statistical computing. Vienna, Austria: R Foundation for Statistical Computing; 2014*. See https://www.R-project.org/.

26. Fish FE. 1998 Comparative kinematics and hydrodynamics of odontocete cetaceans: morphological and ecological correlates with swimming performance. *J. Exp. Biol.* **201**, 2867–2877.
