# Supplementary material for: Effects of body plan evolution on the hydrodynamic drag and energy requirements of swimming in ichthyosaurs
Source: Proc Biol Sci. 2019 Mar 6;286(1898):20182786. doi: 10.1098/rspb.2018.2786 (PMC6458325; doi:10.1098/rspb.2018.2786)
Supplement: Ichthyosaur body sizes [file rspb20182786supp4.pdf]

Data S3. List of ichthyosaur body lengths. Data on total body length (i.e. total length from the tip of the rostrum to the tip of the tail) was collected from the literature or personal examination of specimens. The total length for each genus, shown in bold, has been calculated as the average of the estimations from all available specimens of that genus, and has been used to build the graph in Figure S6.

Genus	species	Total body length (m)	Specimen	Reference	Comments
Cartorhynchus	*lenticarpus*	**0.4**	AGM CH-628-16	Motani et al., 2015[1]	Estimate from reference
Sclerocormus	*parviceps*	**1.6**	AGB6265	Jiang et al., 2016[2]	Estimate from reference
Chaohusaurus	sp.	0.763	AGM I-1 CHS-5 composite	Motani et al., 2014[3]	Fig. 3
Chaohusaurus	sp.	0.99	AGM L-4	Jiang et al., 2016[2]	Estimate from reference
Chaohusaurus	*zhangjiawanenesis*	0.74	WHGMR V26001	Chen et al., 2013[4]	Fig. 2
Chaohusaurus		**0.83**			
Utatsusaurus	*hataii*	2.5	Reconstruction	Motani, 1998[5]	Fig. 2
Cymbospondylus	*petrinus*	9.3	UCMP 9950	Merriam, 1908[6]	Estimate from reference
Cymbospondylus	*buchseri*	5.5	PIMUZ T 4351	Sander, 1989[7]	Estimate from reference
Cymbospondylus		7.4			
Xinminosaurus	*catactes*	2.32	GMPKU-P-1071	Jiang et al., 2008 [8]	Estimate from reference
Mixosaurus	*cornalianus*	0.795		Fröbisch et al., 2013[9]	Estimate from reference
Mixosaurus	*cornalianus*	0.83	PIMUZ T4376	Schmitz et al., 2004[10]	Estimate from reference
Mixosaurus	*cornalianus*	0.9	PIMUZ T2420	Repossi, 1902[11]	Estimate from reference
Mixosaurus	*panxianensis*	0.73	GMPKU-P-1039	Jiang et al., 2006[12]	Estimate from reference
Mixosaurus	*xindianensis*	1	YIGM SPC V-0732	Chen & Cheng, 2010[13]	Estimate from reference
Mixosaurus		**0.85**			
Phalarodon	*callawayi*	**1.50**	CMC VP 7275	Schmitz et al., 2004[10]	Estimate from reference
Besanosaurus	*leptorhynchus*	**5.5**	BES SC 999	Dal Sasso & Pinna, 1996[14]	Estimate from reference
Shonisaurus	*popularis*	13.5	Reconstruction, based in BISP specimens	Kosch, 1990[15]	Fig. 1
Shonisaurus	*sikanniensis*	21	TMP 94.378.2	Nichols & Manabe, 2004 [16]	Estimate from reference
Shonisaurus		**17.25**			
Shastasaurus	*lingae*	8.3	YGMIR SPCV03109	Sander, 2011[17]	Estimate from reference
Shastasaurus	*lingae*	7	YGMIR SPCV03107	Sander, 2011[17]	Estimate from reference
Shastasaurus		**7.65**			
Guizhouichthyosaurus	*tangae*	5.4	TR 00001	Chen & Cheng, 2003[18]	Estimate from reference
Guizhouichthyosaurus	*tangae*	5.2	IVPP V 11853	Shang & Li, 2009[19]	Estimate from reference
Guizhouichthyosaurus	*tangae*	6	Various specimens GNG	Maisch et al., 2006[20]	Estimate from reference
Guizhouichthyosaurus		**5.5**			
Qianichthyosaurus	*zhoui*	1.21	V11838	Li, 1999[21]	Estimate from reference

Qianichthyosaurus	*zhoui*	1.6	V11839, holotype	Li, 1999[21]	Estimate from reference
Qianichthyosaurus	*zhoui*	1.5	CMNH V1412/C1120	Nichols et al., 2002[22]	Estimate from reference
Qianichthyosaurus	*xingyiensis*	1.3	WS2011-46-R1	Yang et al., 2013[23]	Estimate from reference
Qianichthyosaurus		**1.40**			
Suevoleviathan	*integer*	4.3	SMNS 15390	Maxwell, 2018[24]	Estimate from reference
Suevoleviathan	*integer*	2.62	MCZ 1042	Maxwell, 2018[24]	Estimate from reference
Suevoleviathan		**3.46**			
Temnodontosaurus	*platyodon*	6.38	BMNH R 2003	McGowan, 1974[25]	Estimate from reference
Temnodontosaurus	*trigonodon*	7.27	GPIT/RE/9409 (former 1491/13)	McGowan, 1996[26]	Estimate from reference
Temnodontosaurus	*trigonodon*	7.7	SMNS 15950	Maxwell & Vincent, 2016[27]	Estimate from reference
Temnodontosaurus	*trigonodon*	9.09	SMNS 50000	McGowan, 1996[26]	Estimate from reference
Temnodontosaurus	*trigonodon*	7.71	SMNS 52340	McGowan, 1996[26]	Estimate from reference
Temnodontosaurus		**7.63**			
Leptonectes	*tenuirostris*	1.33	BMNH R498	McGowan, 1974[25]	Fig. 12
Leptonectes	*tenuirostris*	1.73	BGS 51236	McGowan & Motani, 2003[28]	Fig. 72
Leptonectes	*tenuirostris*	2.17	M 3564	Personal observation	
Leptonectes	*tenuirostris*	3.8	DLR 002	McGowan, 1993l[29]	Estimate from reference
Leptonectes	*solei*	8	BRSMG Ce 9856	McGowan, 1993[29]	Estimate from reference
Leptonectes	*solei*	5.6	MHN 96270	McGowan, 1993[29]	Fig. 5
Leptonectes		**3.77**			
Hauffiopteryx	*typicus*	1.93	GPIT/RE/9417 (former 1491/4)	Von Huene, 1931[30]	Estimate from reference
Hauffiopteryx	*typicus*	1.55	Composite	Caine & Benton, 2011[31]	Fig. 17
Hauffiopteryx		**1.74**			
Excalibosaurus	*costini*	5.7	ROM 47697	McGowan & Motani, 2003[28]	Fig. 2
Eurhinosaurus	*longirostris*	5.5	Lias ε II,9/10. Frankfurt	Von Huene, 1931[30]	Estimate from reference
Eurhinosaurus	*longirostris*	3.8	MB R 4004	Personal observation	
Eurhinosaurus		**4.65**			
Stenopterygius	*quadriscissus*	3.15	GPIT/RE/9283 (former 43/0219-1)	Von Huene, 1931[30]	Estimate from reference
Stenopterygius	*quadriscissus*	2.3	Lias ε I,2. No 172. Götingen	Von Huene, 1931[30]	Estimate from reference
Stenopterygius	*quadriscissus*	2.5	Lias ε I,2. No 121. St. Petersburg	Von Huene, 1931[30]	Estimate from reference
Stenopterygius	*quadriscissus*	1.8	Lias ε I,2. No 317. Berlin	Von Huene, 1931[30]	Estimate from reference
Stenopterygius	*quadriscissus*	2.5	Lias ε I,2. No 224.	Von Huene,	Estimate from reference

			Berlin	1931[30]	
Stenopterygius	*quadriscissus*	2.9	Lias ε I,2. No 127. Bonn	Von Huene, 1931[30]	Estimate from reference
Stenopterygius	*quadriscissus*	2.7	Lias ε I,2. No 188. Upsala	Von Huene, 1931v[30]	Estimate from reference
Stenopterygius	*quadriscissus*	2.2	Lias ε I,2. No 82	Von Huene, 1931v[30]	Estimate from reference
Stenopterygius	*quadriscissus*	1.6	Lias ε I,2. No 152	Von Huene, 1931[30]	Estimate from reference
Stenopterygius	*quadriscissus*	1.3	Lias ε I,2. No 272	Von Huene, 1931[30]	Estimate from reference
Stenopterygius	*quadriscissus*	2.85	Lias ε II, 4/5. No 276	Von Huene, 1931[30]	Estimate from reference
Stenopterygius	*quadriscissus*	3.1	MHH 1981/33	Maxwell & Vincent, 2016[27]	Estimate from reference
Stenopterygius	*quadriscissus*	1.38	NHM R4086	Personal observation	
Stenopterygius	*quadriscissus*	2.75	SMNS 3775	Von Huene, 1931[30]	Estimate from reference
Stenopterygius	*quadriscissus*	3.04	SMNS 16811	Von Huene, 1931[30]	Estimate from reference
Stenopterygius	*quadriscissus*	2.85	SMNS 50963	Maxwell, 2012[32]	Estimate from reference
Stenopterygius	*quadriscissus*	2.08	SMNS 52036	Maxwell & Vincent, 2016[27]	Estimate from reference
Stenopterygius	*quadriscissus*	2.32	SMF 457	Lingham-Soliar, 2016[33]	Estimate from reference
Stenopterygius	*triscissus*	3.33	SMNS 14846	Maxwell, 2012[32]	Estimate from reference
Stenopterygius	*triscissus*	2.2	GPIT/RE/9416 (former 12/0224-2)	Von Huene, 1931[30]	Estimate from reference
Stenopterygius	*uniter*	3.75	SMNS 17500	Maxwell, 2012[32]	Estimate from reference
Stenopterygius	*uniter*	3.17	SMNS 4865	Von Huene, 1931[30]	Estimate from reference
Stenopterygius	sp.	2.07	Lias ε II,4. No 4	Von Huene, 1931[30]	Estimate from reference
Stenopterygius	sp.	2.36	Lias ε II,10. No 6	Von Huene, 1931[30]	Estimate from reference
Stenopterygius	sp.	2	OUM JZ 163	Von Huene, 1931[30]	Estimate from reference
Stenopterygius	sp.	1.2	Lias ε II,6. No 287	Von Huene, 1931[30]	Estimate from reference
Stenopterygius	sp.	1.9	Lias ε II,6. No 326	Von Huene, 1931[30]	Estimate from reference
Stenopterygius	sp.	1.7	Lias ε II,6. No 105	Von Huene, 1931[30]	Estimate from reference
Stenopterygius	sp.	3.33	Lias ε II,10. No 275	Von Huene, 1931[30]	Estimate from reference
Stenopterygius	sp.	2.5	Lias ε II,11. No 233	Von Huene, 1931[30]	Estimate from reference
Stenopterygius	sp.	3.4	Lias ε II,10. No 132. Upsala	Von Huene, 1931[30]	Estimate from reference
Stenopterygius	sp.	3.7	Lias ε II,10. No 186	Von Huene, 1931[30]	Estimate from reference
Stenopterygius		**2.50**			
Ichthyosaurus	*communis*	0.87	BMNH R1073	McGowan, 1974[25]	Fig. 2

Ichthyosaurus	*communis*	1.58	M3550	Personal observation	
Ichthyosaurus	*somersetensis*	2.7	NHMUK PV OR2013	Massare & Lomax, 2018[34]	Estimate from reference
Ichthyosaurus	*breviceps*	1.21	BMNH 43006	McGowan, 1974[25]	Fig. 7
Ichthyosaurus	*conybeari*	1.3	BGS 956	McGowan, 1974[25]	Estimate from reference
Ichthyosaurus		**1.53**			
Ophthalmosaurus	*icenicus*	4.12	NHMUK PV R3702, R3893 and R4124 composite	Andrews, 1915[35]	Estimate from reference
Ophthalmosaurus	*icenicus*	4.30	GPIT/RE/9410	Personal observation	
Ophthalmosaurus		**4.21**			
Nannopterygius	*enthekiodon*	3.5	BMNH 46497	McGowan & Motani, 2003[28]	Fig. 91
Undorosaurus	*gorodischensis*	**5**	UPM No. EP-II-20 (527)	Arkhangelsky & Zverkov, 2014[36]	Estimate from reference
Cryopterygius	*kristiansenae*	5.5	*PMO 214.578*	Druckenmiller et al. 2012[37]	Estimate from reference
Cryopterygius	kielanae	4	GMUL 3579-81	Tyborowski, 2016[38]	Estimate from reference
Cryopterygius		**4.75**			
Platypterygius	*platydactylus*	4.5	Holotype	McGowan & Motani, 2003[28]	Fig. 95
Platypterygius	*australis*	5.6	F 2453	Wade, 1984[39]	Estimate from reference
Platypterygius	*hercynicus*	4.65	SGS specimen	Kolb and Sander, 2009[40]	Estimate from reference
Platypterygius		**4.92**			
Athabascasaurus	*bituminensis*	**3.5**	TMP 200.29.01	Druckenmiller & Maxwell, 2010[41]	Estimate from reference

References

1. Motani R, Jiang D-Y, Chen G-B, Tintori A, Rieppel O, Ji C, Huang J-D. 2015 A basal ichthyosauriform with a short snout from the Lower Triassic of China. *Nature* **517**, 485–488. (doi:10.1038/nature13866)

2. Jiang D-Y *et al.* 2016 A large aberrant stem ichthyosauriform indicating early rise and demise of ichthyosauromorphs in the wake of the end-Permian extinction. *Scientific Reports* **6**, 26232. (doi:10.1038/srep26232)

3. Motani R, Jiang D, Tintori A, Rieppel O, Chen G. 2014 Terrestrial origin of viviparity in Mesozoic marine reptiles Indicated by Early Triassic embryonic fossils. *PLoS ONE* **9**, e88640. (doi:10.1371/journal.pone.0088640)

4. Chen X, Sander PM, Cheng L, Wang X. 2013 A new Triassic primitive ichthyosaur from Yuanan, South China. *Acta Geologica Sinica* **87**, 672–677. (doi:10.1111/1755-6724.12078)

5. Motani R. 1998 Ichthyosaurian relationships illuminated by new primitive skeletons from Japan. *Nature* **393**, 255–257. (doi:10.1038/30473)

6. Merriam JC. 1908 Triassic Ichthyosauria, with special reference to the American forms. *Memoirs of the University of California* **1**, 1–196.

7. Sander PM. 1989 The large ichthyosaur *Cymbospondylus buchseri*, sp. nov., from the Middle Triassic of Monte San Giorgio (Switzerland), with a survey of the genus in Europe. *Journal of Vertebrate Paleontology* **9**, 163–173. (doi:10.1080/02724634.1989.10011750)

8. Jiang D, Motani R, Hao W, Schmitz L, Rieppel O, Sun Y, Sun Z. 2008 New primitive ichthyosaurian (Reptilia, Diapsida) from the Middle Triassic of Panxian, Guizhou, southwestern China and its position in the Triassic biotic recovery. *Progress in Natural Science* **18**, 1315–1319. (doi:10.1016/j.pnsc.2008.01.039)

9. Fröbisch NB, Fröbisch J, Sander PM, Schmitz L, Rieppel O. 2013 Macropredatory ichthyosaur from the Middle Triassic and the origin of modern trophic networks. *Proceedings of the National Academy of Sciences* **110**, 1393–1397. (doi:10.1073/pnas.1216750110)

10. Schmitz L, Sander PM, Storrs GW, Rieppel O. 2004 New Mixosauridae (Ichthyosauria) from the Middle Triassic of the Augusta Mountains (Nevada, USA) and their implications for mixosaur taxonomy. *Palaeontographica Abteilung A* **270**, 133–162.

11. Repossi E. 1902 Il mixosauro delgi strati Triasici di Besano in Lombardia.pdf. *Atti della Società italiana di scienze naturali e del Museo civico di storia naturale di Milano* **41**, 361–371.

12. Jiang D, Schmitz L, Hao W-C, Sun Y-L. 2006 A new mixosaurid ichthyosaur from the Middle Triassic of China. *Journal of Vertebrate Paleontology* **26**, 60–69. (doi:10.1671/0272-4634(2006)26[60:ANMIFT]2.0.CO;2)

13. Chen X, Cheng L. 2010 A new species of *Mixosaurus* (Reptilia: Ichthyosauria) from the Middle Triassic of Pu'an, Guizhou, China. *Acta Palaeontologica Sinica* **49**, 251–260.

14. Dal Sasso C, Pinna G. 1996 *Besanosaurus leptorhynchus* n.gen. n.sp., a new shastasaurid ichthyosaur from the Middle Triassic of Besano (Lombardy, N. Italy). *Paleontologia Lombarda* **4**, 1–23.

15. Kosch BF. 1990 A revision of the skeletal reconstruction of *Shonisaurus popularis* (Reptilia: Ichthyosauria). *Journal of Vertebrate Paleontology* **10**, 512–514. (doi:10.1080/02724634.1990.10011833)

16. Nicholls EL, Manabe M. 2004 Giant ichthyosaurs of the Triassic—a new species of *Shonisaurus* from the Pardonet Formation (Norian: Late Triassic) of British Columbia. *Journal of Vertebrate Paleontology* **24**, 838–849. (doi:10.1671/0272-4634(2004)024[0838:GIOTTN]2.0.CO;2)

17. Sander PM, Chen X, Cheng L, Wang X. 2011 Short-snouted toothless ichthyosaur from China suggests Late Triassic diversification of suction feeding ichthyosaurs. *PLoS ONE* **6**, e19480. (doi:10.1371/journal.pone.0019480)

18. Chen X, Cheng L. 2003 A new species of large-sized and long-body ichthyosaur from the Late Triassic Guanling biota, Guizhou, China. *Geological Bulletin of China* **22**, 228–235.

19. Shang Q-H, Li C. 2009 On the occurrence of the ichthyosaur *Shastasaurus* in the Guanling biota. *Vertebrata PalAsiatica* **47**, 178–183.

20. Maisch MW, Pan X-R, Sun Z-Y, Cai T, Zhang D-P, Xie J-L. 2006 Cranial osteology of *Guizhouichthyosaurus tangae* (Reptilia: Ichthyosauria) from the Upper Triassic of China. *Journal of Vertebrate Paleontology* **26**, 588–597. (doi:10.1671/0272-4634(2006)26[588:COOGTR]2.0.CO;2)

21. Li C. 1999 Ichthyosaur from Guizhou, China. *Chinese Science Bulletin* **44**, 1329–1333.

22. Nicholls EL, Wei C, Manabe M. 2002 New material of *Qianichthyosaurus* Li, 1999 (Reptilia, Ichthyosauria) from the Late Triassic of southern China, and implications for the distribution of Triassic ichthyosaurs. *Journal of Vertebrate Paleontology* **22**, 759–765. (doi:10.1671/0272-4634(2002)022[0759:NMOQLR]2.0.CO;2)

23. Yang P, Ji C, Jiang D, Motani R, Tintori A, Sun Y, Sun Z. 2013 A new species of *Qianichthyosaurus* (Reptilia Ichthyosauria) from Xingyi Fauna (Ladinian Middle Triassic) of Guizhou. *Acta Scientiarum Naturalium Universitatis Pekinensis* **49**, 1002–1008.

24. Maxwell EE. 2018 Redescription of the 'lost' holotype of *Suevoleviathan integer* (Bronn, 1844) (Reptilia: Ichthyosauria). *Journal of Vertebrate Paleontology* **38**, e1439833. (doi:10.1080/02724634.2018.1439833)

25. McGowan C. 1974 A revision of the longipinnate ichthyosaurs of the Lower Jurassic of England, with descriptions of two new species (Reptilia: Ichthyosauria). *Life sciences contributions, Royal Ontario Museum* **97**, 1–37.

26. McGowan C. 1996 Giant ichthyosaurs of the Early Jurassic. *Canadian Journal of Earth Sciences* **33**, 1011–1021. (doi:10.1139/e96-077)

27. Maxwell EE, Vincent P. 2016 Effects of the early Toarcian Oceanic Anoxic Event on ichthyosaur body size and faunal composition in the Southwest German Basin. *Paleobiology* **42**, 117–126. (doi:10.1017/pab.2015.34)

28. McGowan C, Motani R. 2003 *Handbook of Paleoherpetology: Ichthyopterygia*. München: F. Pfeil.

29. McGowan C. 1993 A new species of large, long-snouted ichthyosaur from the English lower Lias. *Canadian Journal of Earth Sciences* **30**, 1197–1204. (doi:10.1139/e93-101)

30. Von Huene F. 1931 Neue studien über ichthyosaurier aus Holzmaden. *Abhandlungen der Senckenbergischen Naturforschenden Gesellschaft* **42**, 345–382.

31. Caine H, Benton MJ. 2011 Ichthyosauria from the Upper Lias of Strawberry Bank, England. *Palaeontology* **54**, 1069–1093. (doi:10.1111/j.1475-4983.2011.01093.x)

32. Maxwell EE. 2012 New metrics to differentiate species of Stenopterygius (Reptilia: Ichthyosauria) from the Lower Jurassic of southwestern Germany. *Journal of Paleontology* **86**, 105–115. (doi:10.1666/11-038.1)

33. Lingham-Soliar T. 2016 Convergence in thunniform anatomy in lamnid sharks and Jurassic ichthyosaurs. *Integrative and Comparative Biology* **56**, 1323–1336. (doi:10.1093/icb/icw125)

34. Massare JA, Lomax DR. 2018 A taxonomic reassessment of *Ichthyosaurus communis* and *I. intermedius* and a revised diagnosis for the genus. *Journal of Systematic Palaeontology* **16**, 263–277. (doi:10.1080/14772019.2017.1291116)

35. Andrews CW. 1915 I.—Note on a Mounted Skeleton of *Ophthalmosaurus icenicus*, Seeley. *Geological Magazine* **2**, 145–146.

36. Arkhangelsky MS, Zverkov NG. 2014 On a new ichthyosaur of the genus *Undorosaurus*. *Proceedings of the Zoological Institute RAS* **318**, 10.

37. Druckenmiller PS, Hurum JH, Knutsen EM, Nakrem HA. 2012 Two new ophthalmosaurids (Reptilia: Ichthyosauria)- from the Agardhfjellet Formation (Upper Jurassic: Volgian/Tithonian),- Svalbard, Norway. *Norwegian Journal of Geology* **92**, 311–329.

38. Tyborowski D. 2016 A new ophthalmosaurid ichthyosaur species from the Late Jurassic of Owadów-Brzezinki Quarry, Poland. *Acta Palaeontologica Polonica* **61**. (doi:10.4202/app.00252.2016)

39. Wade M. 1984 *Platypterygius australis*, an Australian Cretaceous ichthyosaur. *Lethaia* **17**, 99–113. (doi:10.1111/j.1502-3931.1984.tb01713.x)

40. Kolb C, Sander PM. 2009 Redescription of the ichthyosaur *Platypterygius hercynicus* (Kuhn 1946) from the Lower Cretaceous of Salzgitter (Lower Saxony, Germany). *Palaeontographica Abteilung A: Palaozoologie−Stratigraphie* **288**, 151–192.

41. Druckenmiller PS, Maxwell EE. 2010 A new Lower Cretaceous (lower Albian) ichthyosaur genus from the Clearwater Formation, Alberta, Canada. *Canadian Journal of Earth Sciences* **47**, 1037–1053. (doi:10.1139/E10-028)
